# Variance as a life history outcome: Sensitivity analysis of the contributions of stochasticity and heterogeneity

**DOI:** 10.1016/j.ecolmodel.2019.108856

**Published:** 2020-02-01

**Authors:** Silke van Daalen, Hal Caswell

**Affiliations:** Institute for Biodiversity and Ecosystem Dynamics, University of Amsterdam, PO Box 94248, 1090 GE Amsterdam, The Netherlands

**Keywords:** Variance decomposition, Sensitivity analysis, Individual stochasticity, Heterogeneity, Lifetime reproductive output, Longevity, Life history

## Abstract

Variance in life history outcomes among individuals is a requirement for natural selection, and a determinant of the ecological dynamics of populations. Heterogeneity among individuals will cause such variance, but so will the inherently stochastic nature of their demography. The relative contributions of these variance components – stochasticity and heterogeneity – to life history outcomes are presented here in a general, demographic calculation. A general formulation of sensitivity analysis is provided for the relationship between the variance components and the demographic rates within the life cycle. We illustrate these novel methods with two examples; the variance in longevity within and between frailty groups in a laboratory population of fruit flies, and the variance in lifetime reproductive output within and between initial environment states in a perennial herb in a stochastic fire environment. In fruit flies, an increase in mortality would increase the variance due to stochasticity and reduce that due to heterogeneity. In the plant example, increasing mortality reduces, and increasing fertility increases both variance components. Sensitivity analyses such as these can provide a powerful tool in identifying patterns among life history stages and heterogeneity groups and their contributions to variance in life history outcomes.

## Highlights

• Individuals vary in traits or in luck; both cause variance in life history outcomes.
• The variance components are calculated from a multistate group-stage cohort model.
• Sensitivity analysis shows how variance components relate to demographic parameters.
• Both mortality and fertility affect the variance components.
• Effects depend on life history timing, and the nature and mixture of differences.

## 1 Introduction

The life histories of all species are characterized by variability. No two individuals live the same life, leading to variability in such demographic outcomes as longevity, growth and developmental trajectories, and lifetime reproductive output. Such variance among individuals ultimately determines both the ecological dynamics and the evolutionary potential of species. Lifetime reproductive output is often positively skewed, with many individuals producing few offspring and a long tail of rare individuals producing many offspring (Clutton-Brock, 1988, Newton, 1989). Similarly, longevity varies among individuals, with deaths often peaking at very early life history stages and again at later stages where senescence occurs (Caughley, 1966).

Inter-individual variation in demographic outcomes arises from two sources. One is heterogeneity among individuals; the other is chance differences among homogeneous individuals. We define individuals to be homogeneous if they are, at every stage of life, subject to identical demographic rates (mortality, reproduction, growth, development, movement, etc.). For example, if a set of individuals all experience the same mortality schedule, their trajectories from birth to death differ only when one individual is luckier than the other. The inter-individual variability in lifetime outcomes that arises from such chance processes is called *individual stochasticity* (Caswell, 2009).^1^

On the other hand, if the set of individuals contains groups that, at any stage of life, experience different rates, these differences will contribute to inter-individual variability. Within each group, inter-individual variability is due to stochasticity (because within each group, all individuals experience the same rates), whereas the difference among groups results from heterogeneity in traits or environments linked to the different rates experienced by the different groups.

Heterogeneity is a broad term, encompassing all manners of differences in vital rates among individuals. One might think that heterogeneity is a new concern, but that is far from the case. Demography is, and always has been, a science of heterogeneity. The recognition that individuals of different ages are heterogeneous in mortality and fertility motivated the development of life tables, longevity statistics, stable population theory and the rest of age-classified demography (e.g., Lotka, 1939, Leslie, 1945). The recognition that size, developmental stage, or physiological condition might be more important motivated stage-classified demography (e.g., Lefkovitch, 1965, Metz and Diekmann, 1986, Caswell, 1989).

Within age- or stage-classified demography, we know how to calculate the variance due to individual stochasticity in longevity (Caswell, 2009) and lifetime reproductive output (van Daalen and Caswell, 2017). These analyses, in the form of matrix population models, provide a basis for analyzing the effect of additional sources of heterogeneity, by incorporating them into the demographic state space.

Start by defining a set of individuals that is of interest. Inter-individual variability within this set, in some demographic outcome, will depend on heterogeneity and stochasticity. To make the discussion more concrete, suppose that the set consists of individuals within one age class, say age at birth. Suppose that the individuals in the set are heterogeneous in a property called frailty, which influences the mortality rates experienced at any age.

Suppose further that the outcome of interest is longevity. We divide the set of individuals into groups based on their frailty at birth. The lives of the individuals within each group unfold under the influence of stochasticity. Each group has its own typical outcome.

The variable defining such heterogeneity may remain fixed at its initial value (e.g., genotype, or some kinds of frailty) or may change dynamically through the life cycle (e.g., resources, physiological condition, health; see Table 1). If dynamic, its changes contribute to the stochastic component of the variation.

**Table 1:** Examples of types of individual heterogeneity.

	Internal	External
Fixed	Genotype	Experimental treatments
	Frailty	Social status
	Other (latent) traits	Location

Dynamic	Health	Environmental stochasticity
	Prior state	Density

Matrix population models have been used to quantify the contributions of individual stochasticity and heterogeneity to the variance in demographic outcomes (e.g., Hartemink and Caswell, 2018, Caswell et al., 2018). Some studies have found that stochasticity alone can account for most, or at least a substantial fraction, of the observed variance in lifetime reproductive output of, e.g., nematodes and polychaetes (Caswell, 2011), seabirds (Tuljapurkar et al., 2009, Steiner et al., 2010, Steiner and Tuljapurkar, 2012), birds and mammals (van Daalen, 2015), and humans (van Daalen and Caswell, 2019). In such cases, heterogeneity can be invoked only with additional evidence. One of the best forms of evidence is statistical identification and estimation of the heterogeneity, either as a latent or an observed variable (e.g., Cam et al., 2016, Authier et al., 2017).

Given such an estimate, a quantitative assessment of the contribution of heterogeneity is achieved by incorporating the heterogeneity into the i-state space of the demographic model, resulting in a multistate or age × stage-classified model. These models are constructed using vec-permutation matrix methods (Caswell, 2014, Caswell and Salguero-Gómez, 2013, Caswell et al., 2018). Individuals are classified according to a two-dimensional state space; one dimension describes the progression through the life history (e.g., age, size, developmental stage), and the other dimension describes the movement among heterogeneity “groups”. The groups may represent observable properties (e.g., health, resources, environmental conditions) or unobserved latent variables (e.g., frailty). Methods for estimation of latent variables from individual data is a rapidly growing field of its own (recently reviewed in Gimenez et al. (2018) and Hamel et al. (2018); see also works on mixture models such as McLachlan and Peel (2004), Erişoğlu et al. (2012)).

The multistate model that results from expanding the state space is itself a Markov chain, from which variance in a variety of demographic outcomes can be calculated. We will focus on longevity and lifetime reproductive output. Because the model contains groups within which all individuals experience identical rates, but which differ from each other, it is possible to partition the variance into variance within groups (attributable to individual stochasticity) and variance among the groups (resulting from heterogeneity).

As of this writing, in most cases, only a small fraction of the variance can be attributed to heterogeneity. Hartemink et al. (2017) found that unobserved frailty in the gamma-Gompertz–Makeham mortality model contributed less than 10% of the variance in longevity in several human populations. An analysis of laboratory insect populations found that latent heterogeneity in mortality parameters contributed a median of 35% of the variance in longevity (Hartemink and Caswell, 2018). Jenouvrier et al. (2018) found that unobserved heterogeneity in the southern fulmar contributed 5.9% of the variance in longevity, 3.7% of the variance in age at first reproduction, and 22% of the variance in lifetime reproductive output. Snyder and Ellner (2018) partitioned the variance in a population of kittiwakes and a population of plants, where the heterogeneity was, again, unobserved.^2^ In these studies, heterogeneity could generally explain a small fraction of the longevity and age at first reproduction, and a slightly larger fraction of lifetime reproductive output.

Heterogeneity can also arise from external factors that are directly observable (but possibly neglected). One such set of factors in human populations are lumped under the term socioeconomic status. Status, as measured by income, education, or other metrics, is known to affect life expectancy. However, several analyses have found that only a small fraction (a few percent) of the variance in longevity is due to socioeconomic heterogeneity (Seaman et al., 2019, Caswell, 2019a, Vaupel et al., 2011).

In plants and animals external factors often correspond to environmental conditions. A set of individuals at the same age or stage may be heterogeneous in location, microclimate, resource availability, or disturbance history. That heterogeneity will contribute to variance in the demographic outcome. Environmental conditions may be dynamic, such as fire or resource availability, or fixed, such as microclimate variation in sessile organisms. We provide examples of both fixed and dynamic heterogeneity, in Sections 4 and 5.

By incorporating the heterogeneity, be it fixed or dynamic, into the individual state space, we turn the variance, and its components, into demographic quantities. As such, they depend on the life history characteristics, including the definitions of the state space, the lifespan of the species, whether the heterogeneity affects survival, reproduction, or development (or some combination of these), and the way the individuals are *mixed* among the heterogeneity groups. As with any other demographic quantity, sensitivity analysis is a powerful and essential tool for analyzing this dependence and quantifying how changes in the life history will change variance components.

In this study, we present a general sensitivity analysis of the components of variance in an arbitrary demographic outcome. We will begin by defining a notation for the variance component calculations, and then develop sensitivity formulae. We illustrate the sensitivity analysis with two examples: an age × frailty-classified model for a species of fruit fly, and a stochastic stage × environment model for a perennial herb with a fire-dependent life history.

In the case of the fruit fly, increasing mortality increases the variance due to stochasticity and reduces the variance due to heterogeneity. The effects are strongest at early ages. In the case of the plant, increasing mortality reduces both variance components; increasing fertility increases both components. The effects are strongest at large size classes. The diversity of these responses demonstrates that sensitivity analysis can reveal connections between variance components and the parameters that define the life cycle. They also suggest the value of further theoretical and comparative analyses.

**A note on notation.** Our analysis requires careful mathematical notation to be clear, so we specify our general notation here. Matrices are denoted by upper-case boldface letters (e.g., P), and vectors by lower-case boldface letters (e.g., ρ). Vectors are column vectors by default; $x^{T}$ is the transpose of x. The vector $1_{n}$ is a n×1 vector of ones, $I_{n}$ is the identity matrix of order n, and $e_{i}$ is the ith unit vector, with a 1 in the ith entry and zeros elsewhere. The diagonal matrix with the vector x on the diagonal and zeros elsewhere is denoted D(x). The symbol ∘ denotes the Hadamard, or element-by-element product and ⊗ denotes the Kronecker product. The vec operator vecX stacks the columns of an m×n matrix X into an mn×1 column vector. The vec-permutation matrix $K_{m,n}$ satisfies $\mathrm{vec}\left(X^{T}\right)=K_{m,n}\mathrm{vec}\;X$. In cases where it will help understanding, we indicate the dimension of displayed matrix expressions. The expectation and variance are denoted by E(·) and V(·).

## 2 Variance decomposition in demographic models

To develop our results, we must bring together several different components and methods. We first define the variance components, then the sensitivity of the variance components in general, and then the demographic model from which they can be calculated. Finally, we will show how to make the sensitivity calculations specific by applying them to two examples.

### 2.1 Stages, groups, and outcomes

The population is classified into stages x=1,…,s (age or size classes, developmental stages, etc.) and a set of heterogeneity groups γ=1,…,g (genotype, frailty, environmental condition, etc.). The definition of stages and groups is investigator-specific. For example, from the perspective of a stage-classified model, age is a form of heterogeneity; from the perspective of an age-classified model, the same is true of developmental stage (Caswell et al., 2018).

We are interested in a demographic outcome which we denote by ξ. The properties of the random variable ξ depend on the action of the vital rates over the life cycle, including transitions among stages within the heterogeneity groups, and transitions among heterogeneity groups, to the extent that such transitions are possible.

### 2.2 Conditional means and variances

We define the conditional means and variances of ξ, conditional on group membership, as

(1) $$m_{i}=E\left(\xi |\gamma =i\right),$$

(2) $$v_{i}=V\left(\xi |\gamma =i\right)$$

for i=1,…,g. These conditional values are collected in mean and variance vectors

(3) $$m=\left(\begin{array}{c} m_{1}\\ \vdots \\ m_{g} \end{array}\right)\;v=\left(\begin{array}{c} v_{1}\\ \vdots \\ v_{g} \end{array}\right).$$

The calculation of m and v depends on the life cycle and on the choice of variable ξ.

The distribution of individuals among groups is given by a probability distribution vector π, of dimension g×1. We refer to this as the *mixing distribution* (sensu Frühwirth-Schnatter, 2006). The mixing distribution determines how individuals are distributed among the heterogeneity groups, and thus is one of the determinants of the variance in demographic outcomes.

The mixing distribution is specified at the point in the life cycle from which the demographic outcome is calculated (e.g., at birth or some other chosen stage). The mixing distribution must be specified by the investigator (this is a feature, not a bug). It might be obtained from a model (e.g., the group distribution in the stable population), estimated from data (Hartemink and Caswell, 2018, Jenouvrier et al., 2018), measured from observed covariates (Seaman et al., 2019), or assumed (e.g., a uniform distribution) as a pseudo-experiment.

### 2.3 Variance components

Variance components are calculated from the conditional means and variances. The mean of ξ is the mean, calculated over the distribution π of γ, of the conditional means,

(4) $$E(\xi )=E_{\pi }\left[E(\xi |\gamma )\right]$$

(5) $$=\pi^{T}m.$$

The variance of ξ contains two terms,

(6) $$V(\xi )=E_{\pi }\left[V\left(\xi |\gamma \right)\right]+V_{\pi }\left[E\left(\xi |\gamma \right)\right]$$

(7) $$=\underset{\mathrm{within}}{\underset{︸}{V_{w}(\xi )}}+\underset{\mathrm{between}}{\underset{︸}{V_{b}(\xi )}},$$

where $V_{w}$ is the within-group variance and $V_{b}$ is the between-group variance (e.g., Rényi, 1970, Frühwirth-Schnatter, 2006). The within-group variance is the mean of the conditional variances, with weights specified by π. The between-group variance is the weighted variance of the conditional group means. These variance components capture the effects of heterogeneity and stochasticity. If the groups had identical rates, there would be no heterogeneity, and $V_{b}$ would be zero. If the outcome within each group was totally determined by group membership, there would be no stochasticity, and $V_{w}$ would be zero.

In our notation, the within-group and between-group variances are computed as

(8) $$V_{w}(\xi )=\pi^{T}v,$$

(9) $$V_{b}(\xi )=\pi^{T}\left(m\circ m\right)-{\left(\pi^{T}m\right)}^{2}.$$

The fraction of the total variance contributed by heterogeneity,

(10) $$K=\frac{V_{b}}{V_{b}+V_{w}}$$

is known as the intraclass correlation coefficient in quantitative genetics (Falconer, 1960), and its square root is known as the correlation ratio in probability theory (Rényi, 1970). If ξ is independent of γ, then K=0. If K=1, then ξ is a deterministic function of γ (Rényi, 1970) and individual stochasticity makes no contribution to the variance in ξ.

### 2.4 Sensitivity of the variance components

To find the sensitivity of $V_{b}$, $V_{w}$, and K to changes in demographic parameters we differentiate Eqs. (8) and (9) with respect to m and v. Our methods are an application of matrix calculus; see Caswell (2019b) for a summary of ecological applications and Magnus and Neudecker, 1985, Magnus and Neudecker, 1988 for detailed mathematics.

As always in these applications, we begin by writing the differentials of the variance components in terms of the differentials of the model components. The chain rule then permits us to write the derivative of the variance component with respect to a vector θ of parameters that affect the vital rates. In this paper, we focus on parametric effects on m and v. We will treat the mixing distribution π as fixed, so derivatives with respect to π do not appear.

**Within-group variance.** Differentiating (8) gives

(11) $$dV_{w}=\frac{\partial V_{w}}{\partial v}dv$$

(12) $$=\pi^{T}dv.$$

Let θ be a vector of parameters of interest; the chain rule gives

(13) $$\frac{dV_{w}}{d\theta^{T}}=\pi^{T}\frac{dv}{d\theta^{T}}.$$

**Between-group variance.** The differential of the between-group variance, from (9), is

(14) $$dV_{b}=\frac{\partial V_{b}}{\partial m^{T}}dm$$

(15) $$=\left[2\pi^{T}D(m)-2\pi^{T}m\pi^{T}\right]dm.$$

Once again, dependence on a parameter vector θ yields

(16) $$\frac{dV_{b}}{d\theta^{T}}=\left[2\pi^{T}D(m)-2\pi^{T}m\pi^{T}\right]\frac{dm}{d\theta^{T}}.$$

**Intra-class correlation.** Using Eqs. (13) and (16), it is also possible to differentiate K, as given in (10), with respect to a vector of parameters, giving

(17) $$\frac{dK}{d\theta^{T}}=\left(\frac{1+K}{V_{b}+V_{w}}\right)\frac{dV_{b}}{d\theta^{T}}-\left(\frac{K}{V_{b}+V_{w}}\right)\frac{dV_{w}}{d\theta^{T}}.$$

The expressions (13), (16), and (17) are the droids we are looking for. They require the derivatives of the conditional means and variances with respect to the model parameters. These derivatives in turn depend on the model, the choice of an outcome ξ, and the mixing distribution. To find them, we begin by constructing the model.

## 3 Incorporating heterogeneity: vec-permutation models for stages and groups

In order to calculate both within- and between-group variances, we incorporate both demographic i-states and heterogeneity groups into the demographic model. In this section, we present a general age × stage framework that does so. We use it to develop expressions for the conditional means and variances of the outcome ξ, from which the variance components $V_{b}$, $V_{w}$, and K can be computed. The model provides expressions that can be differentiated with respect to parameters specific to the life history stages and heterogeneity groups.

The multistate model is constructed using the vec-permutation framework, which can incorporate both fixed and dynamic heterogeneity. A complete description of the method, for constructing age × stage models, is given in Caswell et al. (2018), so we do not repeat the details here. When heterogeneity is dynamic; i.e., when individuals can change from one group to another during their lives, it is essential to incorporate the heterogeneity into the state space. When heterogeneity is fixed the vec-permutation approach is equivalent to treating each group independently but it has the advantage of permitting exploration of non-fixed groups.

### 3.1 Model construction

The population composition can be thought of as given by a matrix

(18) $$N=\left(\begin{array}{ccc} n_{11} & \cdots & n_{1s}\\ \vdots & & \vdots \\ n_{g1} & \cdots & n_{\mathrm{g s}} \end{array}\right)$$

in which rows correspond to heterogeneity groups and columns to stages. The population vector $\tilde{\text{n}}$ is obtained by applying the vec operator to N, which results in(19)
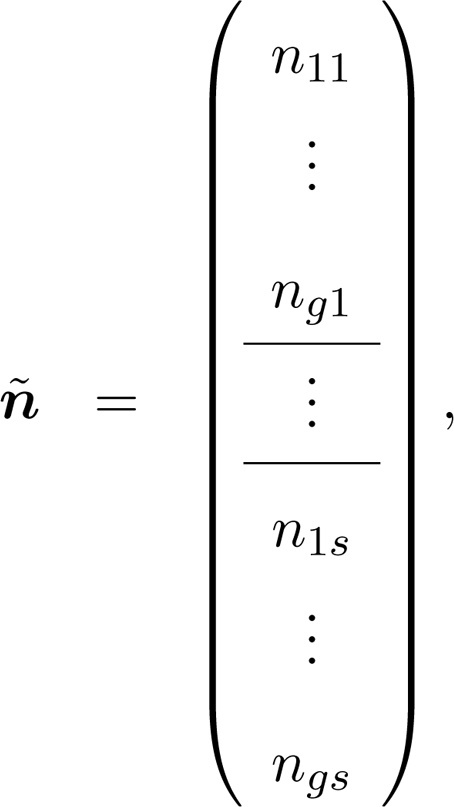
where groups are organized within stages. The population projection matrix is

(20) $$\tilde{\text{A}}=\tilde{\text{U}}+\tilde{\text{F}},$$

where the survival and transition matrix $\tilde{\text{U}}$ and the fertility matrix $\tilde{\text{F}}$ each inherit the group-within-stage block structure of (19). We use the tilde to identify vectors or matrices with this block structure. These matrices are constructed from demographic rates specific to both stages and groups, and capture both kinds of transitions. The s×s matrix $U_{i}$ describes transitions among stages for group i. The g×g matrix $D_{j}$ describes transitions among groups for individuals in stage j. If heterogeneity is fixed, $D_{j}$ is an identity matrix. The s×s matrix $F_{i}$ gives stage-specific fertility for individuals in group i, and the g×g matrix $H_{j}$ allocates offspring newly produced by stage j into their appropriate groups. These matrices are incorporated into gs×gs block diagonal matrices U, D, F, and ℍ. For example,

(21) $$U=\sum_{i=1}^{g}\left(L_{i}U_{i}Q_{i}\right),$$

where $L_{i}$ and $Q_{i}$ are block-construction matrices (Caswell and van Daalen, 2016), given as

(22) $$L_{i}=\left(\begin{array}{c} 0_{(i-1)s\times s}\\ I_{s\times s}\\ 0_{(g-i)s\times s} \end{array}\right),\;Q_{i}=\left(\begin{array}{ccc} 0_{s\times s(i-1)} & I_{s\times s} & 0_{s\times s(g-i)} \end{array}\right)\;i=1,\ldots ,g.$$

Finally, the block-structured projection matrices are given by

(23) $$\tilde{U}=D\;K\;U\;K^{T},$$

(24) $$\tilde{F}=ℍ\;K\;F\;K^{T}.$$

The vec-permutation matrix K permutes the rows and columns in such a way that the orientation defined by (19) is preserved.

### 3.2 Moments of demographic outcomes

We consider a demographic outcome that can be calculated from (and thus inherits the structure of) the multistate Markov chain; we denote this vector outcome by $\tilde{\xi }$. Its entries give the outcome for all gs combinations of stage and group. The model will provide the moments of $\tilde{\xi }$, which in turn provide the mean and variance vectors,(25)
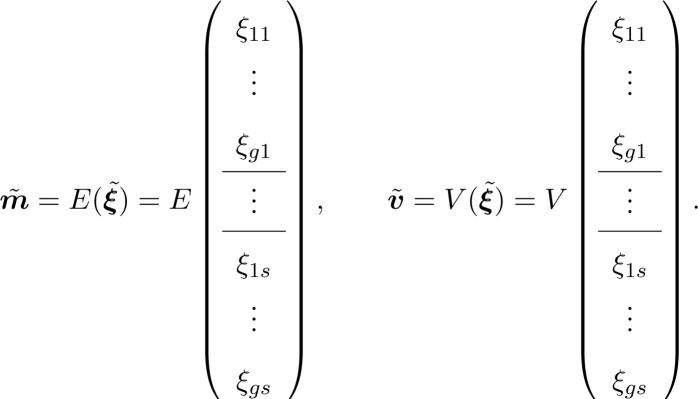
The vectors m and v of the conditional means and variances within the set of interest are obtained from $\tilde{\text{m}}$ and $\tilde{\text{v}}$. Specify a stage j in which our cohort of interest will be initialized. This is often the first stage, or stage at birth, but it could be any stage. Then, the conditional mean and variance vectors in Eq. (3) are given by

(26) $$m=\left(e_{j}^{T}⊗I_{g}\right)\tilde{\text{m}},$$

(27) $$v=\left(e_{j}^{T}⊗I_{g}\right)\tilde{\text{v}},$$

where $e_{j}$ is the jth unit vector of length s; it extracts the jth life history stage and $I_{g}$ keeps all the heterogeneity groups.

The demographic outcome ξ is a function of some set of parameters, collected in a vector θ. So far we have not specified the outcome ξ or how it is calculated. We do so now for two cases, longevity and lifetime reproduction.

**Variance in longevity.** Longevity is the outcome of the mortality hazards faced by an individual as it moves through life history stages and among heterogeneity groups. The mean and variance of longevity are calculated from the multistate fundamental matrix

(28) $$\tilde{\text{N}}={\left(I-\tilde{\text{U}}\right)}^{-1},$$

from which we obtain the vectors of the first and second moments of longevity ($\tilde{\eta }$) as

(29) $${\tilde{\eta }}_{1}^{T}=1_{\mathrm{g s}}^{T}\tilde{\text{N}},$$

(30) $${\tilde{\eta }}_{2}^{T}={\tilde{\eta }}_{1}^{T}(2\tilde{\text{N}}-I_{\mathrm{g s}}).$$

These moment vectors inherit the stage-within-group structure of $\tilde{\text{U}}$. From these, the mean and variance vectors in (25) are

(31) $$\tilde{\text{m}}={\tilde{\eta }}_{1},$$

(32) $$\tilde{\text{v}}={\tilde{\eta }}_{2}-\left({\tilde{\eta }}_{1}\circ {\tilde{\eta }}_{1}\right).$$

**Variance in lifetime reproductive output.** Lifetime reproductive output depends on mortality and fertility for all stages and all heterogeneity groups. The life cycle transitions are described by the Markov chain transition matrix(33)
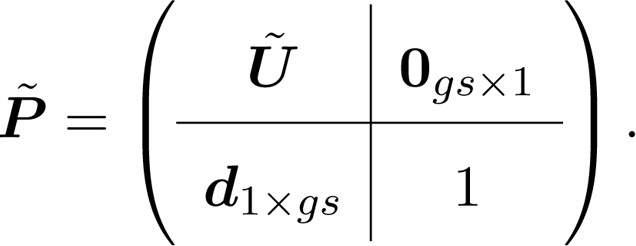
The final state, in the last row of $\tilde{\text{P}}$, is the absorbing state of death. The vector $d_{1\times \mathrm{g s}}$ can be written as a matrix when there are multiple absorbing states, i.e., ${\tilde{\text{M}}}_{\alpha \times \mathrm{g s}}$, where α represents the number of absorbing states.

Reproduction is described as a *reward* that individuals accumulate as they survive and move among the stages of their life cycle (Caswell, 2011, van Daalen and Caswell, 2015, van Daalen and Caswell, 2017, van Daalen and Caswell, 2019). The statistics of reproduction are given by matrices containing the moments of rewards associated with all possible transitions in $\tilde{\text{P}}$; we will be concerned with the first and second moments ${\tilde{\text{R}}}_{1}$ and ${\tilde{\text{R}}}_{2}$. See van Daalen and Caswell (2017) for a general discussion of how these moments might be obtained.

Lifetime reproductive output is the accumulated reproductive reward over the lifetime of an individual. Its first and second moments are

(34) $${\tilde{\rho }}_{1}={\tilde{\text{N}}}^{T}Z{\left(\tilde{\text{P}}\circ {\tilde{\text{R}}}_{1}\right)}^{T}1_{\mathrm{g s}+1},$$

(35) $${\tilde{\rho }}_{2}={\tilde{\text{N}}}^{T}\left[Z{\left(\tilde{\text{P}}\circ {\tilde{\text{R}}}_{2}\right)}^{T}1_{\mathrm{g s}+1}+2{\left(\tilde{\text{U}}\circ {\hat{R}}_{1}\right)}^{T}{\tilde{\rho }}_{1}\right].$$

where Z is a matrix that cleaves off the absorbing state ($Z^{T}$ glues it back on) and ${\hat{R}}_{k}$ is the submatrix of ${\tilde{\text{R}}}_{k}$ without the absorbing state (van Daalen and Caswell, 2017).^3^

The vectors ${\tilde{\rho }}_{1}$ and ${\tilde{\rho }}_{2}$, of dimension g s×1, inherit the block structure of $\tilde{\text{U}}$, and give the (remaining) lifetime reproductive output of individuals in every group, within every stage. In terms of these moments, the vectors containing the multistate means and variances of lifetime reproductive output in (25) are

(36) $$\tilde{\text{m}}={\tilde{\rho }}_{1},$$

(37) $$\tilde{\text{v}}={\tilde{\rho }}_{2}-({\tilde{\rho }}_{1}\circ {\tilde{\rho }}_{1}).$$

### 3.3 Sensitivity analysis

**Fig. 1:**
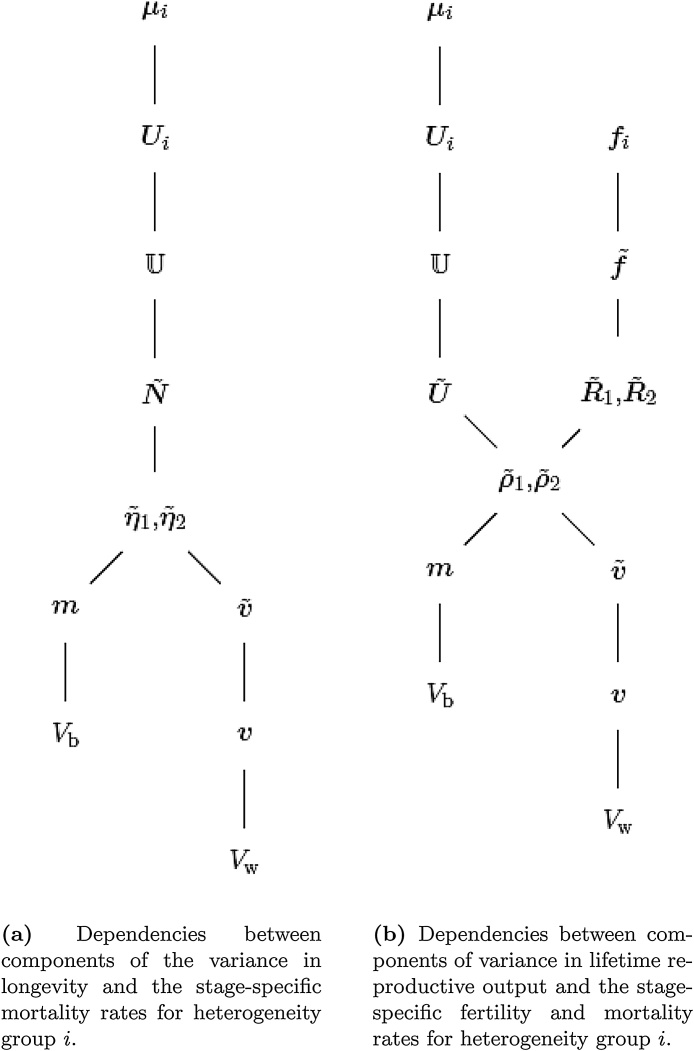
Example dependencies between parameters and variance components in a multistate model.

In order to obtain the sensitivity of the variance components it is useful to consider the paths of dependency between the parameters and the outcome. Fig. 1a shows the links between the mortality schedule of heterogeneity group i and the components of variance in longevity. Fig. 1b shows the links between the fertility and mortality schedules of group i and the components of variance in lifetime reproductive output.

In the paths in Fig. 1a and b, vital rates are first translated into the multistate framework (U and $\tilde{\text{f}}$, respectively), then transformed into the matrices used in the computations ($\tilde{\text{U}}$ and $\tilde{\text{N}}$ for longevity calculations, and $\tilde{\text{U}}$, ${\tilde{\text{R}}}_{1}$, and ${\tilde{\text{R}}}_{2}$ for LRO calculations).

#### 3.3.1 Longevity

The sensitivity equations for the components of variance in longevity, corresponding to the dependencies in Fig. 1a, are

(38) $$\begin{array}{cc} {\left(\frac{dV_{b}}{d\mu^{T}}\right|}_{\mathrm{group}=i} & =\left(\frac{dV_{b}}{dm^{T}}\right)\left(\frac{dm}{d{\tilde{\text{m}}}^{T}}\right)\left(\frac{d\tilde{\text{m}}}{d{\mathrm{vec}}^{T}\tilde{\text{N}}}\right)\left(\frac{d\mathrm{vec}\;\tilde{\text{N}}}{d{\mathrm{vec}}^{T}\tilde{\text{U}}}\right)\\ & \;\times \left(\frac{d\mathrm{vec}\;\tilde{\text{U}}}{d{\mathrm{vec}}^{T}U}\right)\left(\frac{d\mathrm{vec}\;U}{d{\mathrm{vec}}^{T}U_{i}}\right)\left(\frac{d\mathrm{vec}\;U_{i}}{d\mu_{i}^{T}}\right), \end{array}$$

(39) $$\begin{array}{cc} {\left(\frac{dV_{w}}{d\mu^{T}}\right|}_{\mathrm{group}=i} & =\left(\frac{dV_{w}}{dv^{T}}\right)\left(\frac{dv}{d{\tilde{\text{v}}}^{T}}\right)\left(\frac{d\tilde{\text{v}}}{d{\mathrm{vec}}^{T}\tilde{\text{N}}}\right)\left(\frac{d\mathrm{vec}\;\tilde{\text{N}}}{d{\mathrm{vec}}^{T}\tilde{\text{U}}}\right)\\ & \;\times \left(\frac{d\tilde{\text{U}}}{d{\mathrm{vec}}^{T}U}\right)\left(\frac{d\mathrm{vec}\;U}{d{\mathrm{vec}}^{T}U_{i}}\right)\left(\frac{d\mathrm{vec}\;U_{i}}{d\mu_{i}^{T}}\right). \end{array}$$

In these expressions, the mean and variance vectors, $\tilde{\text{m}}$ and $\tilde{\text{v}}$, are the vectors given by (31) and (32).

Eqs. (38) and (39) give the sensitivity of the variance components to the stage-specific mortality schedule within a specified group. The effect of perturbations in multiple groups is obtained by adding these group-specific sensitivities.

Some of the derivatives in Eqs. (38) and (39) are determined by the construction of the matrices, and generally applicable to any model. These are given in Table 2. Others are specific to the life cycle and parameterization under investigation; these are presented in Section 4.

**Table 2:** General derivatives for variance components in longevity.

Derivative	Equation
$\frac{dV_{w}}{dv^{T}}=\pi^{T}$	(8)
$\frac{dV_{b}}{dm^{T}}=2\pi^{T}D(m)-2\pi^{T}m\pi^{T}$	(9)
$\frac{dK}{d\theta^{T}}=\frac{1+K}{V_{b}+V_{w}}\frac{dV_{b}}{d\theta^{T}}-\frac{K}{V_{b}+V_{w}}\frac{dV_{w}}{d\theta^{T}}$	(10)
$\frac{dm}{d\tilde{\text{m}}}=\left(e_{j}^{T}⊗I_{s}\right)$	(26)
$\frac{dv}{d{\tilde{\text{v}}}^{T}}=\left(e_{j}^{T}⊗I_{s}\right)$	(27)
$\frac{d\mathrm{vec}\;\tilde{\text{N}}}{d{\mathrm{vec}}^{T}\tilde{\text{U}}}=\left({\tilde{\text{N}}}^{T}⊗\tilde{\text{N}}\right)$	(28)
$\frac{d\mathrm{vec}\;\tilde{\text{U}}}{d{\mathrm{vec}}^{T}U}=\left(K⊗DK\right)$	(23)
$\frac{d\mathrm{vec}\;U}{d{\mathrm{vec}}^{T}U_{i}}=\left(Q_{i}^{T}⊗L_{i}\right)$	(21)

#### 3.3.2 Lifetime reproductive output

Lifetime reproductive output depends on both fertility and mortality. Following the dependencies illustrated in Fig. 1b, we have the sensitivities of the between-group variance $V_{b}$ to fertility and to mortality,

(40) $$\begin{array}{cc} {\left(\frac{dV_{b}}{df^{T}}\right|}_{\mathrm{group}=i} & =\left(\frac{dV_{b}}{dm^{T}}\right)\left(\frac{dm}{d{\tilde{\text{m}}}^{T}}\right)\left(\frac{d\tilde{\text{m}}}{d{\mathrm{vec}}^{T}{\tilde{\text{R}}}_{1}}\right)\\ & \;\times \left(\frac{d\mathrm{vec}\;{\tilde{\text{R}}}_{1}}{d{\tilde{\text{f}}}^{T}}\right)\left(\frac{d\tilde{\text{f}}}{df_{i}^{T}}\right), \end{array}$$

(41) $$\begin{array}{cc} {\left(\frac{dV_{b}}{d\mu^{T}}\right|}_{\mathrm{group}=i} & =\left(\frac{dV_{b}}{dm^{T}}\right)\left(\frac{dm}{d{\tilde{\text{m}}}^{T}}\right)\left(\frac{d\tilde{\text{m}}}{d{\mathrm{vec}}^{T}\tilde{\text{U}}}\right)\\ & \;\times \left(\frac{d\mathrm{vec}\;\tilde{\text{U}}}{d{\mathrm{vec}}^{T}U}\right)\left(\frac{d\mathrm{vec}\;U}{d{\mathrm{vec}}^{T}U_{i}}\right)\left(\frac{d\mathrm{vec}\;U_{i}}{d\mu_{i}^{T}}\right). \end{array}$$

Note that the fertility vector f affects $V_{b}$ only through ${\tilde{\text{R}}}_{1}$, and the mortality vector μ affects $V_{b}$ only through $\tilde{\text{U}}$. In these and the following equations, $\tilde{\text{m}}$ and $\tilde{\text{v}}$ are the vectors defined in Eqs. (36) and (37).

The sensitivities of the within-group variance $V_{w}$ are

(42) $$\begin{array}{cc} {\left(\frac{dV_{w}}{df^{T}}\right|}_{\mathrm{group}=i} & =\left(\frac{dV_{w}}{dv^{T}}\right)\left(\frac{dv}{d{\tilde{\text{v}}}^{T}}\right)\left(\frac{d\tilde{\text{v}}}{d{\tilde{\text{f}}}^{T}}\right)\left(\frac{d\tilde{\text{f}}}{df_{i}^{T}}\right)\\ & =\left(\frac{dV_{w}}{dv^{T}}\right)\left(\frac{dv}{d{\tilde{\text{v}}}^{T}}\right)\left[\left(\frac{d\tilde{\text{v}}}{d{\mathrm{vec}}^{T}{\tilde{\text{R}}}_{1}}\right)\right)\\ & \;\left(+\left(\frac{d\tilde{\text{v}}}{d{\mathrm{vec}}^{T}{\tilde{\text{R}}}_{2}}\right)\left(\frac{d\mathrm{vec}\;{\tilde{\text{R}}}_{2}}{d{\mathrm{vec}}^{T}{\tilde{\text{R}}}_{1}}\right)\right]\left(\frac{d\mathrm{vec}\;{\tilde{\text{R}}}_{1}}{d{\tilde{\text{f}}}^{T}}\right)\left(\frac{d\tilde{\text{f}}}{df_{i}^{T}}\right), \end{array}$$

(43) $$\begin{array}{cc} {\left(\frac{dV_{w}}{d\mu^{T}}\right|}_{\mathrm{group}=i} & =\left(\frac{dV_{w}}{dv^{T}}\right)\left(\frac{dv}{d{\tilde{\text{v}}}^{T}}\right)\left(\frac{d\tilde{\text{v}}}{d{\mathrm{vec}}^{T}\tilde{\text{U}}}\right)\left(\frac{d\mathrm{vec}\;\tilde{\text{U}}}{d\mu_{i}^{T}}\right)\\ & =\left(\frac{dV_{w}}{dv^{T}}\right)\left(\frac{dv}{d{\tilde{\text{v}}}^{T}}\right)\left(\frac{d\tilde{\text{v}}}{d{\mathrm{vec}}^{T}\tilde{\text{U}}}\right)\\ & \;\times \left(\frac{d\mathrm{vec}\;\tilde{\text{U}}}{d{\mathrm{vec}}^{T}U}\right)\left(\frac{d\mathrm{vec}\;U}{d{\mathrm{vec}}^{T}U_{i}}\right)\left(\frac{d\mathrm{vec}\;U_{i}}{d\mu_{i}^{T}}\right). \end{array}$$

Some of the derivatives of $V_{w}$ with respect to mortality are the same as in the case of longevity, and given in Table 2. Some of the derivatives with respect to fertility are also determined only by the construction of the model and are generally applicable; these are given in Table 3. Those derivatives in (40)–(43) that are specific to the life cycle and the model are given in the case study of Section 5.

**Table 3:** General derivatives for variance components in lifetime reproductive output with respect to fertility.

Derivative	Equation
$\frac{dV_{w}}{dv^{T}}=\pi^{T}$	(8)
$\frac{dV_{b}}{dm^{T}}=2\pi^{T}D(m)-2\pi^{T}m\pi^{T}$	(9)
$\frac{dK}{d\theta^{T}}=\frac{1+K}{V_{b}+V_{w}}\frac{dV_{b}}{d\theta^{T}}-\frac{K}{V_{b}+V_{w}}\frac{dV_{w}}{d\theta^{T}}$	(10)
$\frac{dm}{d\tilde{\text{m}}}=\left(e_{j}^{T}⊗I_{s}\right)$	(26)
$\frac{dv}{d{\tilde{\text{v}}}^{T}}=\left(e_{j}^{T}⊗I_{s}\right)$	(27)
$\frac{d\mathrm{vec}\;{\tilde{\text{R}}}_{2}}{d{\mathrm{vec}}^{T}{\tilde{\text{R}}}_{1}}=\left(I_{{\left(\mathrm{g s}+1\right)}^{2}}+2D(\mathrm{vec}\;{\tilde{\text{R}}}_{1})\right)$	(54)
$\frac{d\mathrm{vec}\;{\tilde{\text{R}}}_{1}}{d{\tilde{\text{f}}}^{T}}=\left(Z^{T}⊗1_{\mathrm{g s}+1}\right)$	(52)
$\frac{d\tilde{\text{f}}}{df_{i}}=KL_{i}$	(51)

## 4 Case study: heterogeneous frailty and variance in longevity

To illustrate sensitivity analysis, we analyze the components of the variance in longevity for an age-classified population with latent heterogeneity groups that affect mortality. We will call this latent variable “frailty,” although it is not exactly frailty *sensu* Vaupel and Yashin (1985) and Vaupel et al. (1979), who used the term to describe a multiplicative hazard effect. The analysis is based on data from an experimental population of the fruit fly *Anastrepha obliqua*, as described in Vaupel et al. (1998). That study reported daily mortality for 134,807 adult female flies.

**Fig. 2:**
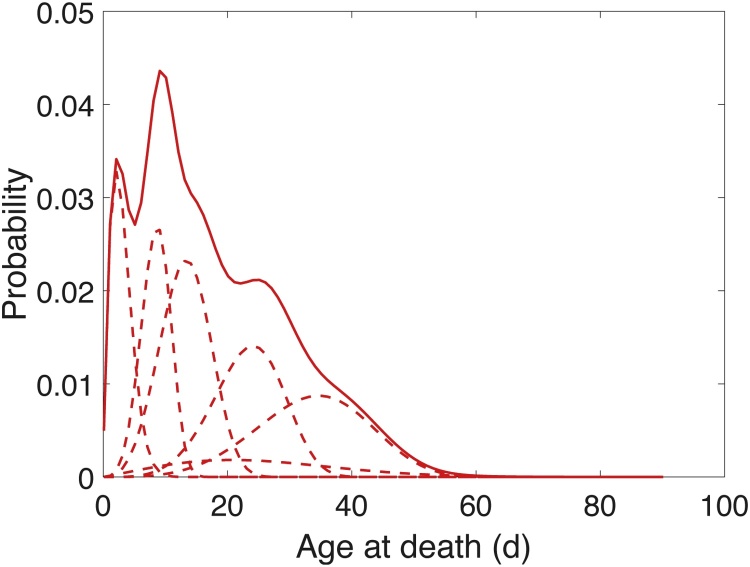
Weibull distributions for age at death for groups of *A. obliqua* females with a different frailty. Dashed lines represent the distributions of age at death for the frailty groups. The solid line is the mixture of these Weibull distributions across groups.

In a search for latent, unobserved heterogeneity, Hartemink and Caswell (2018) fit a mixture of Weibull survival functions to these data using the EM algorithm. AIC methods identified a model with six heterogeneity groups, each with a different set of Weibull parameters and thus a different age schedule of mortality. Fig. 2 shows the distributions of age at death for the six frailty groups and for the mixture of the six groups. All analyses were performed in Matlab (code is available in the supplementary material).

**Fig. 3:**
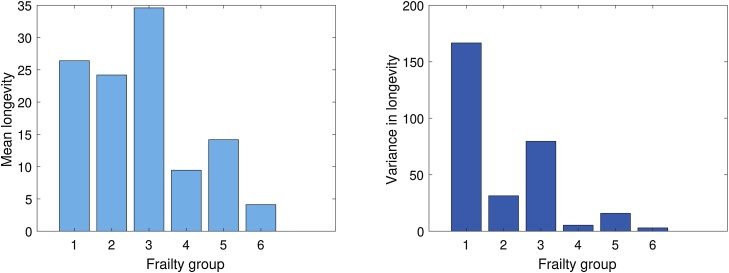
Mean and variance in longevity at eclosion for the different frailty groups in fruit flies.

The mean and variance of longevity were calculated using Eqs. (28)–(32), and are shown in Fig. 3. The first three frailty groups have higher mean longevity, and especially groups 1 and 3 have high variance in longevity as well.

The mixing distribution of newly eclosed adult flies among frailty groups is was estimated as

(44) $$\pi ={\left(\begin{array}{cccccc} 0.06 & 0.20 & 0.20 & 0.16 & 0.24 & 0.14 \end{array}\right)}^{T}.$$

(Hartemink and Caswell, 2018). The variance components, calculated from Eqs. (5), (6), (7), (8), (9), (10), are

$$\begin{array}{c} E_{\pi }(\eta )=18.9,\\ V_{w}=37.3,\\ V_{b}=109.4,\\ V=146.6,\\ K=0.746. \end{array}$$

That is, 75% of the variance in longevity arises from heterogeneity in frailty between individuals, with 25% of the variance due to individual stochasticity. This is a large contribution of heterogeneity relative to other species (Hartemink and Caswell, 2018), which makes it an interesting example on which to perform sensitivity analysis.

The derivatives in Eqs. (38) and (39) that are specific to this case (i.e., specific to an age-classified life cycle, longevity as the dependent variable, mortality as the independent variable) are as follows. Differentiating (29) with respect to $\tilde{\text{N}}$ gives

(45) $$\frac{d\tilde{\text{m}}}{d{\mathrm{vec}}^{T}\tilde{\text{N}}}=\left(I_{\mathrm{g s}}⊗1_{\mathrm{g s}}^{T}\right).$$

Differentiating (32) with respect to $\tilde{\text{N}}$ gives

(46) $$\begin{array}{cc} \frac{d\tilde{\text{v}}}{d{\mathrm{vec}}^{T}\tilde{\text{N}}} & =2{\tilde{\text{N}}}^{T}\left(I_{\mathrm{g s}}⊗1_{\mathrm{g s}}^{T}\right)-\left(I_{\mathrm{g s}}⊗1_{\mathrm{g s}}^{T}\right)\\ & \;+2\left(I_{\mathrm{g s}}⊗{\tilde{\eta }}_{1}^{T}\right)-2D({\tilde{\eta }}_{1})\left(I_{\mathrm{g s}}⊗1_{\mathrm{g s}}^{T}\right). \end{array}$$

Because the model is age-classified, $U_{i}$ is given by

(47) $$U_{i}=Y\circ (1_{s}\sigma_{i}^{T}),$$

where Y is a matrix with ones on the subdiagonal and zeros elsewhere, and the survival vector $\sigma_{i}$ is

(48) $$\sigma_{i}=e^{-\mu_{i}}.$$

Differentiating (47) gives

(49) $$\frac{d\mathrm{vec}\;U_{i}}{d\mu_{i}^{T}}=-D(\mathrm{vec}\;Y)\left(I_{s}⊗1_{s}\right)D(\sigma_{i}).$$

**Fig. 4:**
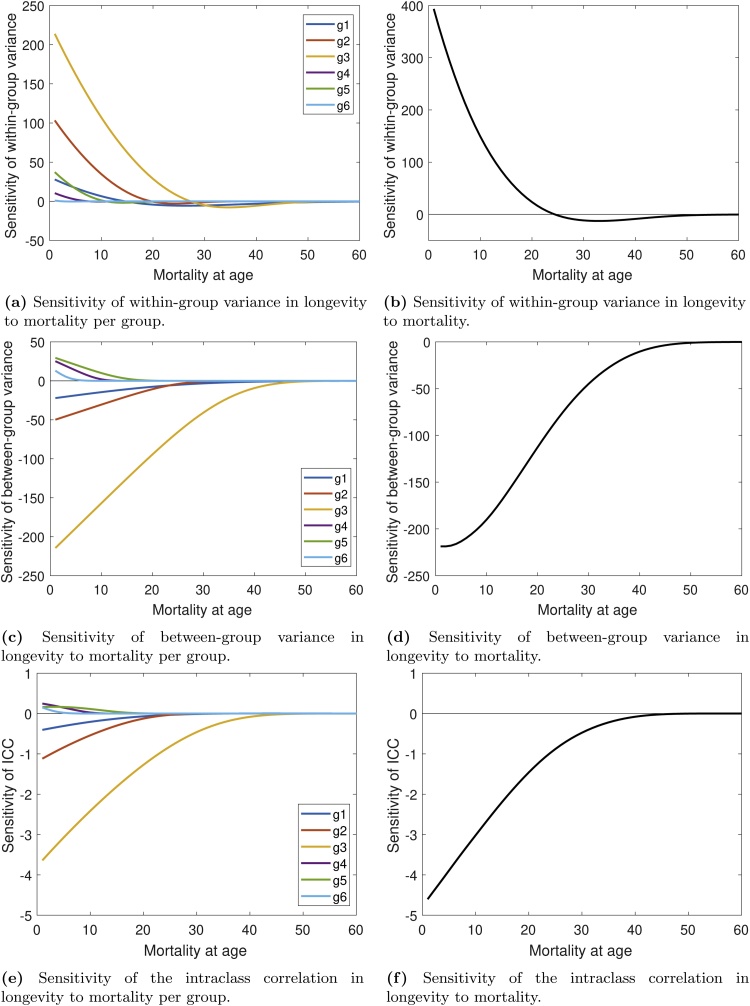
Sensitivity analyses of the variance components in longevity in *A. obliqua* fruit flies. The colored lines in the left column represent a change in the within-group variance (a), the between-group variance (c) and the intraclass correlation, K, (e) resulting from changing mortality in one of the frailty groups. The black line in the right column represents the change in the within-group (b) and between-group (d) variance components, and K (f) for the full population if mortality changed for all groups simultaneously.

The results of the sensitivity analysis are shown in Fig. 4. The left column shows the sensitivity of $V_{w}$ (Fig. 4a), $V_{b}$ (Fig. 4c), and K (Fig. 4e) to changes in age-specific mortality within each group, holding mortality in other groups constant. These are the conditional derivatives in Eqs. (38) and (39). Mortality in group 3 has a strong positive effect on $V_{w}$ and a strong negative effect on $V_{b}$. The effect on K is negative. Overall the effects of group-specific mortality on K are qualitatively similar to the effects on $V_{b}$. Both variance components are most sensitive to changes in mortality at early ages, and practically insensitive to changes after age 20, with the exception of group 3, which happens to have the longest life expectancy (35 days, compared to 19 days for a mixed cohort).

The sensitivities to changes in mortality that affect all frailty groups are shown in Fig. 4b, d and f. All else being equal, an environment with higher overall mortality levels should show a lower fraction of variance in longevity due to heterogeneity. But the details will depend on how heterogeneity affects the vital rates and the mixing of individuals among heterogeneity groups.

The response of $V_{w}$ to mortality at early ages is positive (Fig. 4a). The response becomes negative at late ages; the boundary between “early” and “late” ages differs among groups. The response of $V_{b}$ to changes in mortality is negative when the changes affect long-lived frailty groups, and positive when short-lived frailty groups are affected (Fig. 4c). The pattern of group-specific positive or negative responses of K to changing mortality is similar (Fig. 4e).

## 5 Case study: Environmental stochasticity and variance in lifetime reproductive output

The previous example considered the contribution of fixed heterogeneity to variance in longevity, in an age-classified population. In this case study, we consider the contribution of a dynamic form of heterogeneity to the variance in lifetime reproductive output (LRO), in a stage-classified plant population. The heterogeneity in this case reflects the environment into which the individual is born. Subsequent environmental conditions (fire in this case) and their stochastic dynamics, are incorporated into the individual states.

Dynamic heterogeneity can have diverse effects on variance. Fixed heterogeneity leads to intra-cohort selection, removing the more frail individuals and reducing the eventual variance; see Caswell (2014) for an example. Dynamic movement among heterogeneity groups can counteract this loss of diversity. However, those dynamics also mix individuals among groups. The lower the rate of mixing, the longer individuals will experience the rates of the environment at the starting stage. The higher the rate of mixing between groups, (in the limit, independent and identically distributed transitions), the less persistent will be the effects of environment at birth. Thus dynamic heterogeneity may either increase or reduce the component of variance due to heterogeneity.

*Lomatium bradshawii* is an endangered perennial herb native to grasslands in Oregon and Washington. *L. bradshawii* is adapted to an environment with frequent fires. Reproductive output increases in years where fires take place (Caswell and Kaye, 2001, Caswell, 2011). Caswell and Kaye (2001) presented stage-classified matrix models for *L. bradshawii* in a stochastically varying fire environment. The stages include a yearling class, two classes of vegetative plants, and three classes of reproductive plants, differentiated by size. Here, we use the fertility and survival data for one of the populations (Rose Prairie) to calculate the statistics and variance components of lifetime reproductive output for this species. Analyses were performed in Matlab (code is available in the supplementary material).

Four environmental states were recognized: a fire year, one year since last fire, two years since last fire, and three or more years since the last fire. The dynamics of the fire environment follow a Markov chain with transition matrix

(50) $$D=\left(\begin{array}{cccc} r & r & r & r\\ 1-r & 0 & 0 & 0\\ 0 & 1-r & 0 & 0\\ 0 & 0 & 1-r & 1-r \end{array}\right),$$

where r is the fire frequency. Each of the four fire states has its own set of survival and fertility matrices.

The survival and transition matrix $\tilde{\text{U}}$ is constructed as in Eq. (23), where D contains six copies of the environmental transition matrix D on the diagonal. The transitions among life history stages ($U_{i}$) differ among fire environments. We calculate mean and variance in lifetime reproductive output according to Eqs. (33)–(37).

The moments of lifetime reproductive output in Eqs. (34) and (35) depend on $\tilde{\text{U}}$ and on the reward matrices ${\tilde{\text{R}}}_{1}$ and ${\tilde{\text{R}}}_{2}$. The reward matrices are constructed from environment-specific fertility schedules. Define a vector $f_{i}$ containing the mean stage-specific fertilities in environment i. The multistate vector of fertilities is(51)
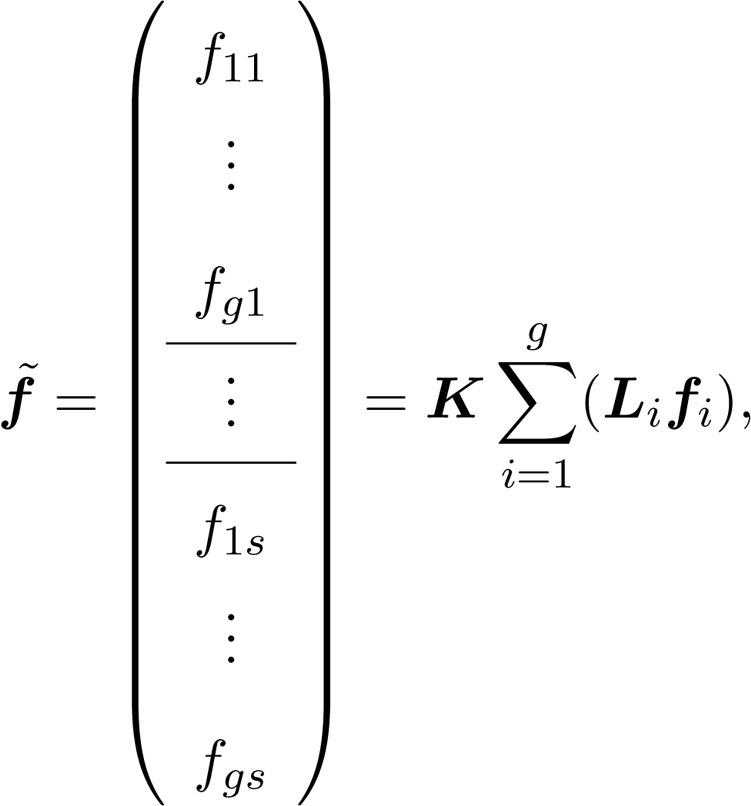
where $L_{i}$ is the block-construction matrix appearing in (22). As in (23), the vec-permutation matrix K permutes the rows and columns so that the structure of $\tilde{\text{f}}$ in (51) matches that of $\tilde{\text{U}}$.

We assume that rewards are obtained when individuals occupy a given state, regardless of their next transition, and that no rewards can be collected by individuals who are dead. These assumptions give the mean reward matrix,(52)
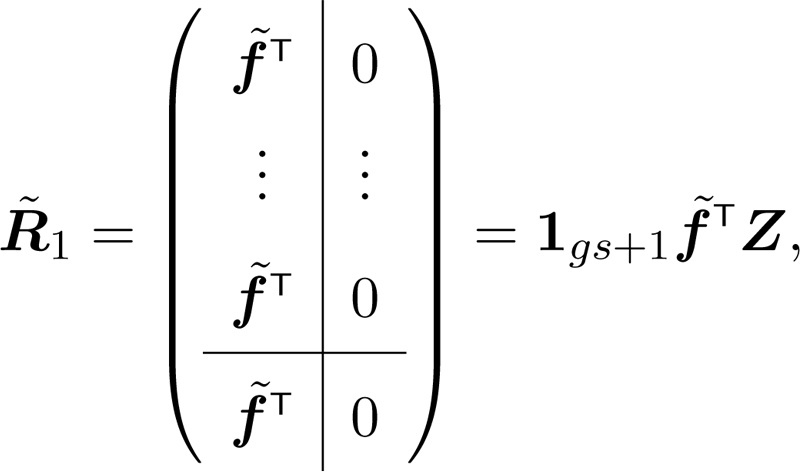
where

(53) $$Z=\left(\begin{array}{c} I|0 \end{array}\right).$$

We obtain the matrix ${\tilde{\text{R}}}_{2}$ of second moments of rewards by assuming that individual reproduction follows a Poisson distribution, so that

(54) $${\tilde{\text{R}}}_{2}={\tilde{\text{R}}}_{1}+({\tilde{\text{R}}}_{1}\circ {\tilde{\text{R}}}_{1}).$$

See Caswell (2011) and van Daalen and Caswell (2017) for discussion of other ways that ${\tilde{\text{R}}}_{2}$ could be obtained.

**Fig. 5:**
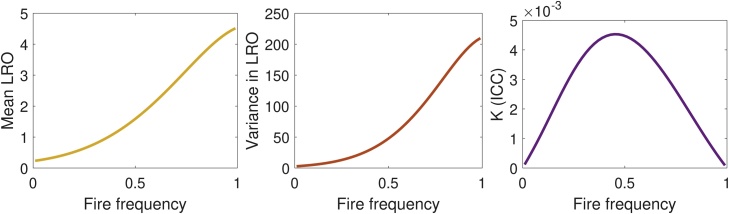
Mean and variance in lifetime reproductive output, and the contribution of environmental heterogeneity to variance in LRO in an endangered perennial herb for changing fire frequencies.

The statistics of LRO depend on the fire frequency r (Fig. 5). Both mean and variance in LRO increase with increasing fire frequency. Despite the strong effect of the fire environment on all the vital rates, the intraclass correlation K is small for this model; environmental heterogeneity contributes less than 0.5% of the variance in LRO. If we continue the analysis at r=0.49, a scenario roughly corresponding to the maximum contribution of heterogeneity, the stochastic, within-environment component of the variance dominates;

$$\begin{array}{c} E_{\pi }(\rho )=1.5,\\ V_{w}=45.2,\\ V_{b}=0.2,\\ V=45.4,\\ K=0.004. \end{array}$$

For sensitivity analysis we keep the fire frequency set to r=0.49. The stable distribution of environments is given by the right eigenvector of the environmental matrix D in Eq. (50). This provides the mixing distribution as

(55) $$\pi ={\left(\begin{array}{cccc} 0.49 & 0.25 & 0.13 & 0.13 \end{array}\right)}^{T}.$$

Other possibilities for mixing distributions have been suggested by Hernandez-Suarez et al. (2012).

The sensitivities of lifetime reproductive output to changes in mortality and fertility within each group (i.e., each environment) are calculated using Eqs. (40)–(43), following the dependencies shown in Fig. 1b. We first investigate the sensitivity of the variance components to fertility. Most of the derivatives are once again independent of the life cycle structure and the dependent and independent variables of interest. These are collected in Table 3.

Differentiating (36) with respect to $\tilde{\text{f}}$ gives

(56) $$\frac{d\tilde{\text{m}}}{d{\tilde{\text{f}}}^{T}}={\tilde{\text{N}}}^{T}\left(1_{\mathrm{g s}+1}⊗Z\right)K_{1}D(\mathrm{vec}\;\tilde{\text{P}})\frac{d\mathrm{vec}\;{\tilde{\text{R}}}_{1}}{d{\tilde{\text{f}}}^{T}}.$$

Differentiating (37) with respect to $\tilde{\text{f}}$ gives

(57) $$\begin{array}{cc} \frac{d\tilde{\text{v}}}{d{\tilde{\text{f}}}^{T}} & ={\tilde{\text{N}}}^{T}\left[\left(1_{\mathrm{g s}+1}⊗Z\right)K_{1}D\left(\mathrm{vec}\;\tilde{\text{P}}\right)\frac{d\mathrm{vec}\;{\tilde{\text{R}}}_{2}}{d{\tilde{\text{f}}}^{T}}\right)\\ & \;\left(+2\left({\tilde{\rho }}_{1}^{T}⊗I\right)K_{2}D(\mathrm{vec}\;\tilde{\text{U}})\left(Z⊗Z\right)\frac{d\mathrm{vec}\;{\tilde{\text{R}}}_{1}}{d{\tilde{\text{f}}}^{T}}\right)\\ & \;\left(+2{\left(\tilde{\text{U}}\circ {\hat{R}}_{1}\right)}^{T}\frac{d\tilde{\text{m}}}{d{\tilde{\text{f}}}^{T}}\right]-2D({\tilde{\rho }}_{1})\frac{d\tilde{\text{m}}}{d{\tilde{\text{f}}}^{T}}, \end{array}$$

where $K_{1}$ and $K_{2}$ are vec-permutation matrices (Magnus and Neudecker, 1979),

(58) $$K_{1}=K_{\left(\mathrm{g s}+1),(\mathrm{g s}+1\right)},$$

(59) $$K_{2}=K_{\mathrm{g s},\mathrm{g s}}.$$

The steps required to obtain (56) and (57) are presented in van Daalen and Caswell (2017) and in the supplementary material.

**Fig. 6:**
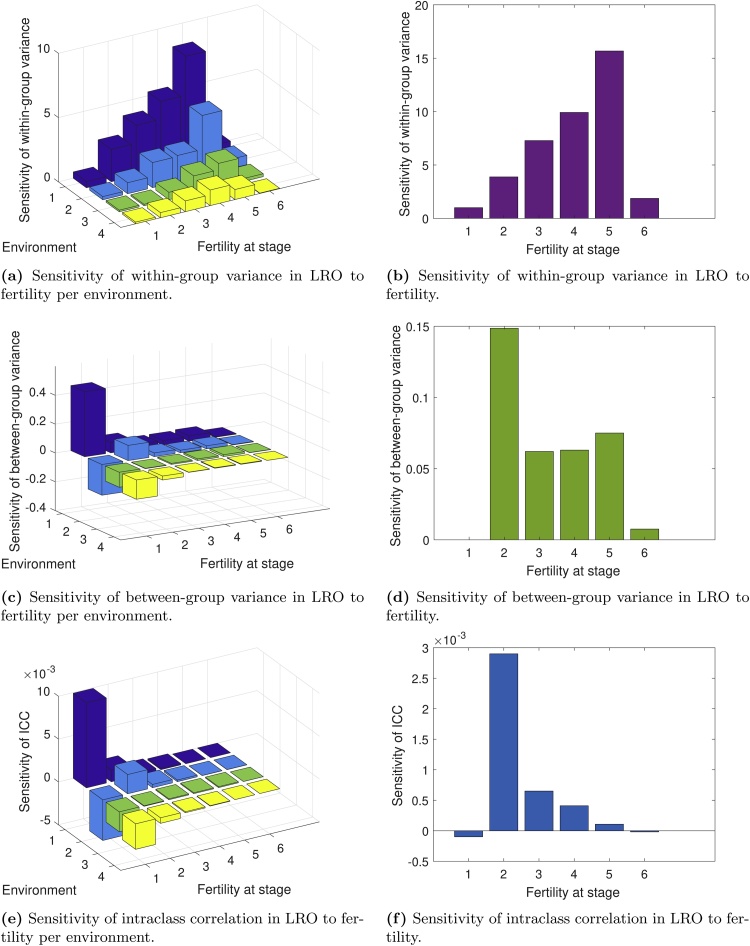
Sensitivity analyses of the variance components of lifetime reproductive output in *L. bradshawii* starting life in the yearling stage. The left-hand panels represent sensitivity of within-group variance (a), between-group variance (c) and the intraclass correlation K (e) to changes in stage-specific fertility in each environment. The panels on the right represent sensitivity of within-group variance (b), between-group variance (d), and intaclass correlation K (f) to changes in fertility in all environments simultaneously.

The sensitivities of $V_{w}$, $V_{b}$, and K to fertility are shown in Fig. 6. The sensitivities of the within-group variance $V_{w}$ to fertility (Fig. 6a and b) increase with stage, up to stage 5. This pattern holds whether fertility is changed within one environment (Fig. 6a) or in all environments (Fig. 6b).

The between-group variance $V_{b}$ is most sensitive to fertility in stage 1 (Fig. 6c), with an increase in fertility in a fire year raising $V_{b}$, whereas in the non-fire-years it lowers $V_{b}$. In later stages fertility has only small positive or negative effects on the between-group variance, depending on the environment. When fertility is increased in all environments, $V_{b}$ increases (Fig. 6d). The net effect of increasing fertility in stage 1 is zero because it increases LRO equally in every environment, as there is no chance of dying differentially before entering stage 1. Therefore the difference among groups does not change.

The intraclass correlation K is most sensitive to fertility in stage 1. Whether the response is positive or negative depends on the stage-environment combination (Fig. 6e).

As is always the case, sensitivity analysis happily reports the results of changes that are hypothetical. In this case, some changes in fertility are impossible, as, for example, stage 1 is a pre-reproductive stage, and increasing fertility at that stage is an interesting thought experiment rather than a potential biological reality. At the final three stages, where reproduction takes place, the effect of changing fertility is far greater for $V_{w}$ than $V_{b}$, but the fraction of variance due to heterogeneity does not change greatly.

Lifetime reproductive output depends on mortality as well as fertility. The sensitivities of the variance components to mortality are given in Eqs. (41) and (43). Most of the pieces of these derivatives are given in Tables 2 and 3 . Unlike (47) for insects, $U_{i}$ for the plants is a function of growth and survival, as in

(60) $$U_{i}=G_{i}\Sigma_{i},$$

(61) $$\Sigma_{i}=I\circ (1_{s}\sigma_{i}^{T}),$$

(62) $$\sigma_{i}=e^{-\mu_{i}}.$$

The sensitivity of $U_{i}$ with respect to $\mu_{i}$ is then

(63) $$\frac{d\mathrm{vec}\;U_{i}}{d\mu_{i}^{T}}=-\left(I⊗G_{i}\right)D(\mathrm{vec}\;I)\left(I_{s}⊗1_{s}\right)D(\sigma_{i}).$$

The derivatives of $\tilde{\text{m}}$ and $\tilde{\text{v}}$ with respect to the transition matrix $\tilde{\text{U}}$ are still needed. They are

(64) $$\begin{array}{cc} \frac{d\tilde{\text{m}}}{d{\mathrm{vec}}^{T}\tilde{\text{U}}} & ={\tilde{\text{N}}}^{T}\left[\left(1_{\mathrm{g s}+1}^{T}⊗Z\right)K_{1}D(\mathrm{vec}\;{\tilde{\text{R}}}_{1})\left(C_{1}-C_{2}\left(I⊗1_{\mathrm{g s}}^{T}\right)\right)\right)\\ & \;\left(+\left({\tilde{\rho }}_{1}^{T}⊗I\right)K_{2}\right], \end{array}$$

(65) $$\begin{array}{cc} \frac{d\tilde{\text{v}}}{d{\mathrm{vec}}^{T}\tilde{\text{U}}} & ={\tilde{\text{N}}}^{T}\left[\left(1_{\mathrm{g s}+1}^{T}⊗Z\right)K_{1}D(\mathrm{vec}\;{\tilde{\text{R}}}_{2})\left(C_{1}-C_{2}\left(I⊗1_{\mathrm{g s}}^{T}\right)\right)\right)\\ & \;\left(+2\left({\tilde{\rho }}_{1}^{T}⊗I\right)K_{2}D(\mathrm{vec}\;{\hat{R}}_{1})+2{\left(\tilde{\text{U}}\circ {\hat{R}}_{1}\right)}^{T}\frac{d\tilde{\text{m}}}{d{\mathrm{vec}}^{T}\tilde{\text{U}}}\right)\\ & \;\left(+\left({\tilde{\rho }}_{2}^{T}⊗I\right)K_{2}\right]-2D({\tilde{\rho }}_{1})\frac{d\tilde{\text{m}}}{d{\mathrm{vec}}^{T}\tilde{\text{U}}}, \end{array}$$

where $C_{1}$ and $C_{2}$ are

(66) $$C_{1}=\left(\begin{array}{c} I_{\mathrm{g s}\times \mathrm{g s}}\\ 0_{1\times \mathrm{g s}} \end{array}\right)⊗\left(\begin{array}{c} I_{\mathrm{g s}\times \mathrm{g s}}\\ 0_{1\times \mathrm{g s}} \end{array}\right),$$

(67) $$C_{2}=\left(\begin{array}{c} I_{\mathrm{g s}\times \mathrm{g s}}\\ 0_{1\times \mathrm{g s}} \end{array}\right)⊗\left(\begin{array}{c} 0_{\mathrm{g s}\times 1}\\ I_{1\times 1} \end{array}\right).$$

The steps required to obtain (64) and (65) are presented in van Daalen and Caswell (2017) and in the supplementary material.

**Fig. 7:**
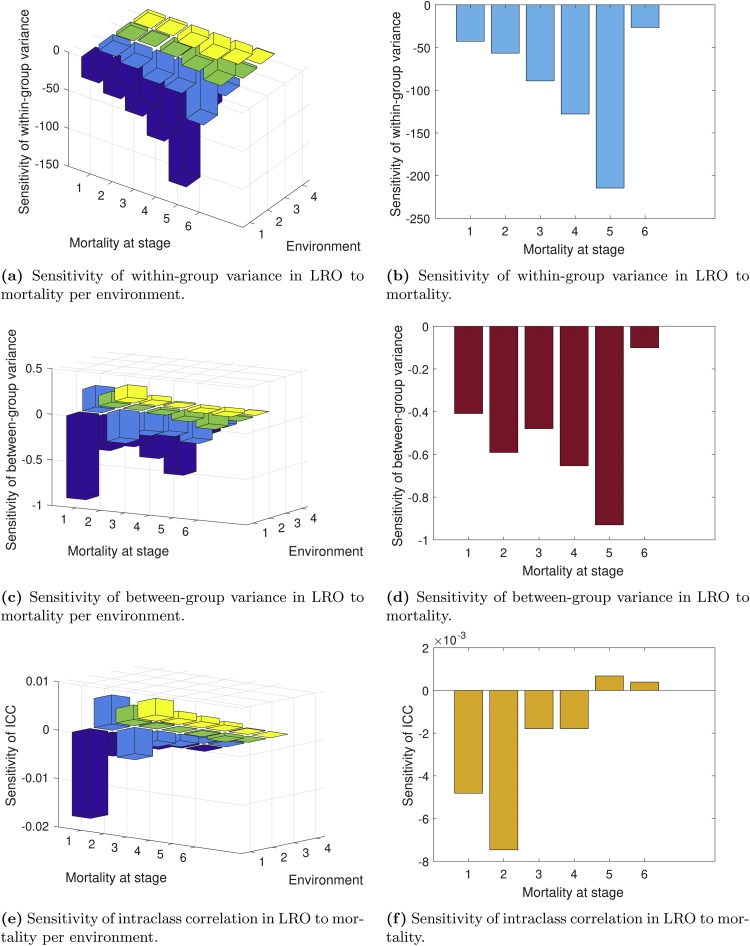
Sensitivity analyses of the variance components of lifetime reproductive output in *L. bradshawii* starting life in the yearling stage. The left-hand panels represent sensitivity of within-group variance (a), between-group variance (c) and the intraclass correlation K (e) to changes in stage-specific mortality in each environment. The panels on the right represent sensitivity of within-group variance (b), between-group variance (d), and intaclass correlation K (f) to changes in mortality in all environments simultaneously.

The sensitivities of $V_{w}$, $V_{b}$, and K to mortality are shown in Fig. 7. Increasing mortality in any environment reduces the within-group variance $V_{w}$ (Fig. 7a). The effects on $V_{b}$ and K depend on the stage-environment combination, but the effects are small (Figs. 7c and e). Increasing mortality in a fire year decreases $V_{b}$, as does increasing mortality in stages 4 and 5.

When mortality increases in all environments, both $V_{w}$ (Fig. 7b) and $V_{b}$ (Fig. 7d) are reduced. The effects on K are small, and mostly negative (Fig. 7f).

## 6 Discussion

Variance components are central to the discussion of the relative contribution of heterogeneity and stochasticity to life history outcomes. Heterogeneity — differences among individuals that lead to differences in the vital rates they experience — is expressed as variance between groups. Individual stochasticity — differences in outcome among individuals experiencing the same rates — appears as variance within groups. The important question is how much each of these sources contributes to the variance.

The results in Sections 2.4 and 3 provide a flexible method for decomposing variance, applicable across life history outcomes, types of heterogeneity, life cycles, sets of vital rates, and mixing distributions. This flexibility is a consequence of formulating the life cycle as a multistate matrix model (Caswell et al., 2018).

The within-group and between-group components of variance depend on the life cycle. As in other parts of demography, sensitivity analysis is useful to explore this dependence (Caswell, 2019b). We present a method for the sensitivity analysis of the variance components to life history parameters and illustrate the approach with two examples: variance in longevity due to heterogeneous frailty in a fruit fly, and variance in lifetime reproductive output due to environmental conditions in an herbaceous plant in a stochastic fire environment.

The fraction of variance explained by heterogeneity differs between these two examples. In the fruit fly, 75% of the variance in longevity is due to heterogeneity in initial frailty. In *L. bradshawii*, only 0.4% of the variance in LRO is due to heterogeneity in initial environmental conditions. Both examples are extreme compared to previous studies, but in different directions. About 5–10% of the variance in longevity is due to frailty in humans (Hartemink et al., 2017). About 5% is due to socio-economic heterogeneity in humans (Seaman et al., 2019). A median of 35% is due to frailty in laboratory insect populations (Hartemink and Caswell, 2018), and about 6% due to unobserved heterogeneity in the Southern Fulmar (Jenouvrier et al., 2018). Studies of lifetime reproductive output have reported contributions of 22% due to unobserved heterogeneity in Southern Fulmars (Jenouvrier et al., 2018), and 39% due to ‘quality’ in kittiwakes (Snyder and Ellner, 2018).

Snyder and Ellner (2018) show that for some analytical models incorporating stochasticity and heterogeneity, stochasticity determines a substantial fraction of the variation even as they vary the values for survival and fertility. In addition, they show that increasing variability in survival and fertility generally increased the contribution of stochasticity. The sensitivity results for the two examples analyzed here partly agree with their result, with the added benefit of disentangling at which ages or stages, and which heterogeneity groups within- and between-group variances are most sensitive to fertility and mortality.

In the fruit fly example, the sensitivity of $V_{w}$ to mortality is positive up to some critical age. The sensitivity of $V_{b}$ is negative at these ages, intuitively leading to a decrease in K with increasing mortality. However, if mortality at early ages increased only for the most ‘frail’ groups of fruit flies, the result would be an increase in K.

In the case of LRO in *L. bradshawii*, the sensitivity of both variance components to fertility is positive, and to mortality is negative. Although K is a simple function of $V_{b}$ and $V_{w}$, the sensitivity of K is not necessarily a simple result of the sensitivities for the variance components. The *Lomatium* example illustrates this, although sensitivity of K in this case is always small.

The sensitivity patterns, the direction and magnitude of the sensitivities of the variance components and K, differ between the two examples. The fact that, in the fruit fly, increasing mortality changes the within-group and between-group components of variance in opposite directions suggests some kind of trade-off, but this is not observed in the example for *Lomatium*. Whether this difference is a result of the structure of the models, the type of heterogeneity, the choice in mixing distribution, or the demographic outcome under investigation, is a question that can only be answered by additional analyses. More studies of variance components of different species across the tree of life would be indispensable in the search for broad patterns.

The mixing distribution π is a key ingredient in variance decomposition, because it defines the initial distribution of individuals among heterogeneity groups. We have treated it as a fixed parameter; a useful extension would be to include the sensitivity of demographic outcomes to π in the analysis. We expect that its relative influence would vary with the type of heterogeneity.

The term “heterogeneity” applies to a wide range of types of differences among individuals. In this paper, we included an example in which an unobserved, internal, fixed set of differences (frailty, in the broad sense) affected the mortality schedule (see Table 1). We also analyzed an example in which the heterogeneity is an observed, external, and dynamic set of differences due to the fire environment. The differences between the vital rates in the fire environments are substantial, yet the between-group variance component is very small. This reflects the dynamic, rather than fixed, nature of the fire environment. Individuals starting life in one environment experience the other environments, decreasing the contribution of the differences in initial environment over the lifetime. Whether this is a general result for dynamic heterogeneity is a question that can only be answered with additional studies.

Additional examples of dynamic heterogeneity and environmental differences, but also different kinds of fixed heterogeneity, and dynamic, sequential heterogeneity groups, will be valuable to elucidate how heterogeneity contributes to the variance in life history outcomes. Many studies include the effects of certain covariates on vital rate estimates; these covariates, if incorporated into a demographic model, might provide the heterogeneity information required for variance analysis.

Life history outcomes are often highly variable among individuals. Especially for fitness components such as lifetime reproductive output, it is important to account for variance due to individual stochasticity, on which selection cannot act (Steiner and Tuljapurkar, 2012, van Daalen and Caswell, 2019). Indeed, individual stochasticity might slow selection by obscuring variance due to genetic heterogeneity (Steiner and Tuljapurkar, 2012). Sensitivity analysis of the variance components to the vital rates is a necessary tool to identify the contributions of heterogeneity and stochasticity to life history outcomes.

## Footnotes

1 It is important to be clear how our terminology relates to other terms independently introduced to describe the same phenomena. Caswell and collaborators refer to the stochastic differences in outcomes from the same vital rates as *individual stochasticity* (e.g., Caswell, 2009, Caswell, 2011, Caswell and Vindenes, 2018, Caswell and Zarulli, 2018), as distinct from heterogeneity. Tuljapurkar and Steiner and collaborators have called the stochastic component *dynamic heterogeneity* and distinguished it from “fixed heterogeneity” (e.g., Tuljapurkar et al., 2009, Steiner et al., 2010, Steiner and Tuljapurkar, 2012). As will become clear later, we want to be able to distinguish fixed and dynamic versions of heterogeneity, which is difficult to do using Tuljapurkar and Steiner's terminology. However, their dynamic heterogeneity corresponds exactly to our individual stochasticity. More recently, Snyder and Ellner have introduced the much more familiar term *luck* for what we call individual stochasticity.

2 Observability is, of course, partly a function of the data and how they are collected, and could change if some new measurement was developed. Unobserved heterogeneity poses statistical challenges to estimation, but there is no fundamental difference between the analysis of observed and unobserved sources of heterogeneity.

3 Two forms of notation collide in this quantity. van Daalen and Caswell (2017) use ${\tilde{\rho }}_{k}$ to denote the vector of kth moments of lifetime reproductive output, with the tilde indicating that absorbing state[s] have been excluded, because it is known a priori that the dead do not reproduce. In this paper, however, the tilde denotes a multistate quantity, with stage-within-group structure. To avoid confusion, we have here adopted the latter convention for $\tilde{\rho }$ as well.
