# Supplementary material for: Variance as a life history outcome: Sensitivity analysis of the contributions of stochasticity and heterogeneity
Source: Ecol Modell. 2020 Feb 1;417:108856. doi: 10.1016/j.ecolmodel.2019.108856 (PMC7015279; doi:10.1016/j.ecolmodel.2019.108856)
Supplement: Supplementary file 1 [file mmc1.pdf]

A Derivations

In order to give some more intuition on the steps involved in deriving the sensitivity formulas, we show here the necessary steps to derive the sensitivities of mean and variance in LRO with respect to fertility and mortality. We start by providing the general derivative of mean LRO (Section A.1), followed by the variance in LRO (Section A.2). Then we show how to get the specific formulas for derivatives with respect to mortality and fertility (Section A.3). Many of these calculations appear in more general form in van Daalen & Caswell (2017).

A.1 Start from the top: Mean lifetime reproductive output

Mean lifetime reproductive output is given by

$$\tilde{\boldsymbol{\rho}}_1 = \tilde{\boldsymbol{N}}^{\mathsf{T}} \boldsymbol{Z} \left(\tilde{\boldsymbol{P}} \circ \tilde{\boldsymbol{R}}_1 \right)^{\mathsf{T}} \mathbf{1}_{gs+1}, \tag{A-1}$$

which we rewrite as the following to make life a bit easier for ourselves;

$$\underbrace{\left(\tilde{\boldsymbol{N}}^{\mathsf{T}}\right)^{-1} \tilde{\boldsymbol{\rho}}_1}_{A} = \underbrace{\boldsymbol{Z} \left(\tilde{\boldsymbol{P}} \circ \tilde{\boldsymbol{R}}_1 \right)^{\mathsf{T}} \mathbf{1}_{gs+1}}_{B}. \tag{A-2}$$

The main steps of sensitivity analysis are to differentiate the expression, then apply the vec operator, and substitute and simplify where necessary. We will do this for both sides of the equation (A-2). Differentiate the left-hand side of the equation to obtain

$$dA = d\left[\left(\tilde{\boldsymbol{N}}^{\mathsf{T}}\right)^{-1}\right] \tilde{\boldsymbol{\rho}}_1 + \left(\tilde{\boldsymbol{N}}^{\mathsf{T}}\right)^{-1} d\tilde{\boldsymbol{\rho}}_1. \tag{A-3}$$

Substituting

$$\left(\tilde{\boldsymbol{N}}^{\mathsf{T}}\right)^{-1} = \left(\boldsymbol{I} - \tilde{\boldsymbol{U}}^{\mathsf{T}}\right) \tag{A-4}$$

yields

$$dA = -\left(d\tilde{\boldsymbol{U}}^{\mathsf{T}}\right) \tilde{\boldsymbol{\rho}}_1 + \left(\boldsymbol{I} - \tilde{\boldsymbol{U}}^{\mathsf{T}}\right) d\tilde{\boldsymbol{\rho}}_1. \tag{A-5}$$

Applying the vec operator gives

$$d\mathrm{vec}\, A = -\mathrm{vec}\, \left[\left(d\tilde{\boldsymbol{U}}^{\mathsf{T}}\right) \tilde{\boldsymbol{\rho}}_1\right] + \mathrm{vec}\, \left[\left(\boldsymbol{I} - \tilde{\boldsymbol{U}}^{\mathsf{T}}\right) d\tilde{\boldsymbol{\rho}}_1\right]. \tag{A-6}$$

We want to apply Roth's theorem,

$$\mathrm{vec}\, (\boldsymbol{ABC}) = (\boldsymbol{C}^{\mathsf{T}} \otimes \boldsymbol{A})\, \mathrm{vec}\, \boldsymbol{B}, \tag{A-7}$$

to extract $\boldsymbol{B}$, in this case the term we are differentiating. We can do this by writing an identity matrix on the "empty" side of the target term (e.g. $\left(d\tilde{\boldsymbol{U}}^{\mathsf{T}}\right) \tilde{\boldsymbol{\rho}}_1 = \boldsymbol{I} \left(d\tilde{\boldsymbol{U}}^{\mathsf{T}}\right) \tilde{\boldsymbol{\rho}}_1$, so that

$$d\mathrm{vec}\, A = -\left(\tilde{\boldsymbol{\rho}}_1^{\mathsf{T}} \otimes \boldsymbol{I}\right) d\mathrm{vec}\, \left(\tilde{\boldsymbol{U}}^{\mathsf{T}}\right) + \left(\boldsymbol{I} - \tilde{\boldsymbol{U}}^{\mathsf{T}}\right) d\tilde{\boldsymbol{\rho}}_1. \tag{A-8}$$

The final step involves writing $\mathrm{vec}\, \left(\tilde{\boldsymbol{U}}^{\mathsf{T}}\right)$ in terms of $\mathrm{vec}\, \tilde{\boldsymbol{U}}$, which requires the vec-permutation matrix $\boldsymbol{K}$,

$$d\mathrm{vec}\, A = -\left(\tilde{\boldsymbol{\rho}}_1^{\mathsf{T}} \otimes \boldsymbol{I}\right) \boldsymbol{K}_2 d\mathrm{vec}\, \tilde{\boldsymbol{U}} + \left(\boldsymbol{I} - \tilde{\boldsymbol{U}}^{\mathsf{T}}\right) d\tilde{\boldsymbol{\rho}}_1. \tag{A-9}$$

In this section we will use several vec-permutation matrices of different sizes, we list them here for reference;

$$\boldsymbol{K} = \boldsymbol{K}_{g,s} \qquad gs \times gs \tag{A-10}$$
$$\boldsymbol{K}_1 = \boldsymbol{K}_{(gs+1),(gs+1)} \qquad (gs+1)^2 \times (gs+1)^2 \tag{A-11}$$
$$\boldsymbol{K}_2 = \boldsymbol{K}_{gs,gs}. \qquad (gs)^2 \times (gs)^2 \tag{A-12}$$

Differentiating the right-hand side of (A-2) gives

$$dB = \boldsymbol{Z}\left(d\tilde{\boldsymbol{P}} \circ \tilde{\boldsymbol{R}}_1\right)^{\mathsf{T}} \mathbf{1} + \boldsymbol{Z}\left(\tilde{\boldsymbol{P}} \circ d\tilde{\boldsymbol{R}}_1\right)^{\mathsf{T}} \mathbf{1}. \tag{A-13}$$

Applying the vec operator (and Roth's theorem, and the vec-permutation matrix) yields

$$d\text{vec}\, B = \left(\mathbf{1}^{\mathsf{T}} \otimes \boldsymbol{Z}\right) \boldsymbol{K}_1 \text{vec}\left(d\tilde{\boldsymbol{P}} \circ \tilde{\boldsymbol{R}}_1\right) + \left(\mathbf{1}^{\mathsf{T}} \otimes \boldsymbol{Z}\right) \boldsymbol{K}_1 \text{vec}\left(\tilde{\boldsymbol{P}} \circ d\tilde{\boldsymbol{R}}_1\right). \tag{A-14}$$

The vec operator is applied to a Hadamard product using the following rule;

$$\text{vec}\,(\boldsymbol{A} \circ \boldsymbol{B}) = \mathcal{D}(\boldsymbol{A})\,\text{vec}\,(\boldsymbol{B}) + \mathcal{D}(\boldsymbol{B})\,\text{vec}\,(\boldsymbol{A}). \tag{A-15}$$

Applying this rule to (A-14) yields

$$d\text{vec}\, B = \left(\mathbf{1}^{\mathsf{T}} \otimes \boldsymbol{Z}\right) \boldsymbol{K}_1 \left[\mathcal{D}\left(\text{vec}\, \tilde{\boldsymbol{R}}_1\right) d\text{vec}\, \tilde{\boldsymbol{P}} + \mathcal{D}\left(\text{vec}\, \tilde{\boldsymbol{P}}\right) d\text{vec}\, \tilde{\boldsymbol{R}}_1\right]. \tag{A-16}$$

As shown in equation (33), $\tilde{\boldsymbol{P}}$ is a block-structured matrix built from $\tilde{\boldsymbol{U}}$ and $\boldsymbol{d}_{1\times gs}$ (and some zeros and ones). More generally, the Markov chain $\tilde{\boldsymbol{P}}$ is written as

$$\tilde{\boldsymbol{P}} = \left(\begin{array}{c|c} \tilde{\boldsymbol{U}} & \mathbf{0} \\ \hline \tilde{\boldsymbol{M}} & \boldsymbol{I} \end{array}\right). \tag{A-17}$$

According to Caswell & van Daalen (2016), the vec operator applied to $\tilde{\boldsymbol{P}}$ can be written as follows

$$d\text{vec}\, \tilde{\boldsymbol{P}} = \boldsymbol{C}_1 d\text{vec}\, \tilde{\boldsymbol{U}} + \boldsymbol{C}_2 d\text{vec}\, \tilde{\boldsymbol{M}}, \tag{A-18}$$

where

$$\boldsymbol{C}_1 = \begin{pmatrix} \boldsymbol{I}_{gs} \\ \mathbf{0}_{\alpha\times gs} \end{pmatrix} \otimes \begin{pmatrix} \boldsymbol{I}_{gs} \\ \mathbf{0}_{\alpha\times gs} \end{pmatrix} \tag{A-19}$$

$$\boldsymbol{C}_2 = \begin{pmatrix} \boldsymbol{I}_{gs} \\ \mathbf{0}_{\alpha\times gs} \end{pmatrix} \otimes \begin{pmatrix} \mathbf{0}_{gs\times\alpha} \\ \boldsymbol{I}_{\alpha} \end{pmatrix}, \tag{A-20}$$

where α is the number of absorbing states. When $\alpha = 1$, the mortality matrix $\tilde{\boldsymbol{M}}$ can be obtained from $\tilde{\boldsymbol{U}}$ as

$$\tilde{\boldsymbol{M}} = \mathbf{1}^{\mathsf{T}} - \mathbf{1}^{\mathsf{T}}\tilde{\boldsymbol{U}}. \tag{A-21}$$

In this case, equation (A-18) further simplifies to

$$d\text{vec}\, \tilde{\boldsymbol{P}} = \left[\boldsymbol{C}_1 - \boldsymbol{C}_2\left(\boldsymbol{I}_{gs} \otimes \mathbf{1}_{gs}^{\mathsf{T}}\right)\right] d\text{vec}\, \tilde{\boldsymbol{U}}. \tag{A-22}$$

From (A-2), $d\text{vec}\, A = d\text{vec}\, B$. Setting (A-9) for $d\text{vec}\, A$ equal to (A-16) for $d\text{vec}\, B$ gives

$$-\left(\tilde{\boldsymbol{\rho}}_1^{\mathsf{T}} \otimes \boldsymbol{I}\right) \boldsymbol{K}_2 d\text{vec}\, \tilde{\boldsymbol{U}} + \left(\boldsymbol{I} - \tilde{\boldsymbol{U}}^{\mathsf{T}}\right) d\tilde{\boldsymbol{\rho}}_1 = \left(\mathbf{1}^{\mathsf{T}} \otimes \boldsymbol{Z}\right) \boldsymbol{K}_1 \left[\mathcal{D}\left(\text{vec}\, \tilde{\boldsymbol{R}}_1\right) d\text{vec}\, \tilde{\boldsymbol{P}} + \mathcal{D}\left(\text{vec}\, \tilde{\boldsymbol{P}}\right) d\text{vec}\, \tilde{\boldsymbol{R}}_1\right]. \tag{A-23}$$

Solving for $d\tilde{\boldsymbol{\rho}}_1$ and substituting $\left(\boldsymbol{I} - \tilde{\boldsymbol{U}}\right)^{\mathsf{T}} = \left(\tilde{\boldsymbol{N}}^{\mathsf{T}}\right)^{-1}$ gives

$$d\tilde{\boldsymbol{\rho}}_1 = \tilde{\boldsymbol{N}}^{\mathsf{T}} \left[\left(\mathbf{1}^{\mathsf{T}} \otimes \boldsymbol{Z}\right) \boldsymbol{K}_1 \left[\mathcal{D}\left(\text{vec}\, \tilde{\boldsymbol{R}}_1\right) d\text{vec}\, \tilde{\boldsymbol{P}} + \mathcal{D}\left(\text{vec}\, \tilde{\boldsymbol{P}}\right) d\text{vec}\, \tilde{\boldsymbol{R}}_1\right] + \left(\tilde{\boldsymbol{\rho}}_1^{\mathsf{T}} \otimes \boldsymbol{I}\right) \boldsymbol{K}_2 d\text{vec}\, \tilde{\boldsymbol{U}}\right]. \tag{A-24}$$

This is the general formula for the sensitivity of mean LRO to the underlying matrices. From here, one can decide which of the parameters in the matrices is of interest, which will be discussed in Section A.3. Let us first obtain the general derivative of the variance in LRO.

A.2 Tackling the beast: Variance in LRO

The variance in lifetime reproductive output is given by

$$\tilde{\boldsymbol{v}} = \tilde{\boldsymbol{\rho}}_2 - (\tilde{\boldsymbol{\rho}}_1 \circ \tilde{\boldsymbol{\rho}}_1). \tag{A-25}$$

Differentiating this and applying the rule for veccing[1] Hadamard products as in (A-15) yields

$$d\tilde{\boldsymbol{v}} = d\tilde{\boldsymbol{\rho}}_2 - 2\mathcal{D}(\tilde{\boldsymbol{\rho}}_1)\, d\tilde{\boldsymbol{\rho}}_1. \tag{A-26}$$

Simple enough. We know $d\tilde{\boldsymbol{\rho}}_1$ from the previous section. All that remains is to obtain the derivative of the second moment of LRO, where the second moment is given by

$$\tilde{\boldsymbol{\rho}}_2 = \tilde{\boldsymbol{N}}^{\mathsf{T}} \left[\boldsymbol{Z} \left(\tilde{\boldsymbol{P}} \circ \tilde{\boldsymbol{R}}_2 \right)^{\mathsf{T}} \mathbf{1}_{gs+1} + 2 \left(\tilde{\boldsymbol{U}} \circ \hat{\boldsymbol{R}}_1 \right)^{\mathsf{T}} \tilde{\boldsymbol{\rho}}_1 \right]. \tag{A-27}$$

Using the same trick as in Section A.1, we write this as

$$\underbrace{\left(\tilde{\boldsymbol{N}}^{\mathsf{T}} \right)^{-1} \tilde{\boldsymbol{\rho}}_2}_{A} = \underbrace{\boldsymbol{Z} \left(\tilde{\boldsymbol{P}} \circ \tilde{\boldsymbol{R}}_2 \right)^{\mathsf{T}} \mathbf{1}_{gs+1}}_{B} + \underbrace{2 \left(\tilde{\boldsymbol{U}} \circ \hat{\boldsymbol{R}}_1 \right)^{\mathsf{T}} \tilde{\boldsymbol{\rho}}_1}_{C}. \tag{A-28}$$

Differentiating term A follows the same steps as we performed in (A-3)-(A-9). The only difference is that the term we solve for here is $\tilde{\boldsymbol{\rho}}_2$;

$$d\text{vec}\, A = -\left(\tilde{\boldsymbol{\rho}}_2^{\mathsf{T}} \otimes \boldsymbol{I} \right) \boldsymbol{K}_2 d\text{vec}\, \tilde{\boldsymbol{U}} + \left(\boldsymbol{I} - \tilde{\boldsymbol{U}}^{\mathsf{T}} \right) d\tilde{\boldsymbol{\rho}}_2. \tag{A-29}$$

Differentiating term B follows the same steps we took earlier in (A-13)–(A-16), except term B in equation (A-28) contains the matrix $\tilde{\boldsymbol{R}}_2$ instead of $\tilde{\boldsymbol{R}}_1$;

$$d\text{vec}\, B = \left(\mathbf{1}^{\mathsf{T}} \otimes \boldsymbol{Z} \right) \boldsymbol{K}_1 \left[\mathcal{D}\left(\text{vec}\, \tilde{\boldsymbol{R}}_2 \right) d\text{vec}\, \tilde{\boldsymbol{P}} + \mathcal{D}\left(\text{vec}\, \tilde{\boldsymbol{P}} \right) d\text{vec}\, \tilde{\boldsymbol{R}}_2 \right]. \tag{A-30}$$

Differentiating term C gives

$$dC = 2 \left[\left(d\tilde{\boldsymbol{U}} \circ \hat{\boldsymbol{R}}_1 \right)^{\mathsf{T}} \tilde{\boldsymbol{\rho}}_1 + \left(\tilde{\boldsymbol{U}} \circ d\hat{\boldsymbol{R}}_1 \right)^{\mathsf{T}} \tilde{\boldsymbol{\rho}}_1 + \left(\tilde{\boldsymbol{U}} \circ \hat{\boldsymbol{R}}_1 \right)^{\mathsf{T}} d\tilde{\boldsymbol{\rho}}_1 \right]. \tag{A-31}$$

Applying the vec operator (and Roth's theorem, the vec-permutation matrix, and the rule for veccing Hadamards) then yields

$$d\text{vec}\, C = 2 \left(\tilde{\boldsymbol{\rho}}_1 \otimes \boldsymbol{I} \right) \boldsymbol{K}_2 \left[\mathcal{D}\left(\text{vec}\, \hat{\boldsymbol{R}}_1 \right) d\text{vec}\, \tilde{\boldsymbol{U}} + \mathcal{D}\left(\text{vec}\, \tilde{\boldsymbol{U}} \right) d\text{vec}\, \hat{\boldsymbol{R}}_1 \right] + 2 \left(\tilde{\boldsymbol{U}} \circ \hat{\boldsymbol{R}}_1 \right)^{\mathsf{T}} d\tilde{\boldsymbol{\rho}}_1. \tag{A-32}$$

Despite the fact that it will not be fun to look at, solving for $\tilde{\boldsymbol{\rho}}_2$ gives

$$\begin{aligned} d\tilde{\boldsymbol{\rho}}_2 \;=\; & \tilde{\boldsymbol{N}}^{\mathsf{T}} \left[\left(\mathbf{1}^{\mathsf{T}} \otimes \boldsymbol{Z} \right) \boldsymbol{K}_1 \left(\mathcal{D}\left(\text{vec}\, \tilde{\boldsymbol{R}}_2 \right) d\text{vec}\, \tilde{\boldsymbol{P}} + \mathcal{D}\left(\text{vec}\, \tilde{\boldsymbol{P}} \right) d\text{vec}\, \tilde{\boldsymbol{R}}_2 \right) \right. \\ & + 2 \left(\tilde{\boldsymbol{\rho}}_1 \otimes \boldsymbol{I} \right) \boldsymbol{K}_2 \left[\mathcal{D}\left(\text{vec}\, \hat{\boldsymbol{R}}_1 \right) d\text{vec}\, \tilde{\boldsymbol{U}} + \mathcal{D}\left(\text{vec}\, \tilde{\boldsymbol{U}} \right) d\text{vec}\, \hat{\boldsymbol{R}}_1 \right] + 2 \left(\tilde{\boldsymbol{U}} \circ \hat{\boldsymbol{R}}_1 \right)^{\mathsf{T}} d\tilde{\boldsymbol{\rho}}_1 \\ & \left. + \left(\tilde{\boldsymbol{\rho}}_2^{\mathsf{T}} \otimes \boldsymbol{I} \right) \boldsymbol{K}_2 d\text{vec}\, \tilde{\boldsymbol{U}} \right]. \end{aligned} \tag{A-33}$$

Fortunately, we can simplify as we get more specific.

A.3 Pick your side: Mortality or fertility

Now that we have formulas (A-24), (A-33), and (A-26), we can differentiate our way down to the parameters we are interested in. Although it is possible that a single (vector of) parameter[s] influences both mortality and fertility simultaneously, in our examples we investigate the effect of a change in these parameter sets separately.

[1] For convenience, we will verb the heck out of the adjective "vec", to mean "applying the vec operator to". Deal with it.

A.3.1 Mortality

When our parameter set of interest is mortality, some of the terms in equations (A-24) and (A-33) will drop out since they do not depend on mortality. We know that $d\text{vec}\,\tilde{\boldsymbol{U}}$, and by extension $d\text{vec}\,\tilde{\boldsymbol{P}}$, depend on mortality through the transition rates between states. $d\text{vec}\,\tilde{\boldsymbol{R}}_1$ and $d\text{vec}\,\tilde{\boldsymbol{R}}_2$, on the other hand, depend on the moments of reproduction, and therefore on fertility. By writing $d\text{vec}\,\tilde{\boldsymbol{P}}$ as in (A-22), the sensitivities of $\tilde{\boldsymbol{\rho}}_1$ and $\tilde{\boldsymbol{\rho}}_2$ with respect to mortality are obtained from (A-24) and (A-33) as

$$\begin{aligned}\frac{d\tilde{\boldsymbol{\rho}}_1}{d\boldsymbol{\mu}_i^{\mathsf{T}}} &= \tilde{\boldsymbol{N}}^{\mathsf{T}}\Bigg[\left(\mathbf{1}^{\mathsf{T}}\otimes\boldsymbol{Z}\right)\boldsymbol{K}_1\mathcal{D}\left(\text{vec}\,\tilde{\boldsymbol{R}}_1\right)\left[\boldsymbol{C}_1-\boldsymbol{C}_2\left(\boldsymbol{I}_{gs}\otimes\mathbf{1}_{gs}^{\mathsf{T}}\right)\right]\frac{d\text{vec}\,\tilde{\boldsymbol{U}}}{d\boldsymbol{\mu}_i^{\mathsf{T}}} \\ &\quad + \left(\tilde{\boldsymbol{\rho}}_1^{\mathsf{T}}\otimes\boldsymbol{I}\right)\boldsymbol{K}_2\frac{d\text{vec}\,\tilde{\boldsymbol{U}}}{d\boldsymbol{\mu}_i^{\mathsf{T}}}\Bigg] \qquad \text{(A-34)}\\ \frac{d\tilde{\boldsymbol{\rho}}_2}{d\boldsymbol{\mu}_i^{\mathsf{T}}} &= \tilde{\boldsymbol{N}}^{\mathsf{T}}\Bigg[\left(\mathbf{1}^{\mathsf{T}}\otimes\boldsymbol{Z}\right)\boldsymbol{K}_1\mathcal{D}\left(\text{vec}\,\tilde{\boldsymbol{R}}_2\right)\left[\boldsymbol{C}_1-\boldsymbol{C}_2\left(\boldsymbol{I}_{gs}\otimes\mathbf{1}_{gs}^{\mathsf{T}}\right)\right]\frac{d\text{vec}\,\tilde{\boldsymbol{U}}}{d\boldsymbol{\mu}_i^{\mathsf{T}}} \\ &\quad +2\left(\tilde{\boldsymbol{\rho}}_1\otimes\boldsymbol{I}\right)\boldsymbol{K}_2\mathcal{D}\left(\text{vec}\,\hat{\boldsymbol{R}}_1\right)\frac{d\text{vec}\,\tilde{\boldsymbol{U}}}{d\boldsymbol{\mu}_i^{\mathsf{T}}}+2\left(\tilde{\boldsymbol{U}}\circ\hat{\boldsymbol{R}}_1\right)^{\mathsf{T}}\frac{d\tilde{\boldsymbol{\rho}}_1}{d\boldsymbol{\mu}_i^{\mathsf{T}}} \\ &\quad + \left(\tilde{\boldsymbol{\rho}}_2^{\mathsf{T}}\otimes\boldsymbol{I}\right)\boldsymbol{K}_2\frac{d\text{vec}\,\tilde{\boldsymbol{U}}}{d\boldsymbol{\mu}_i^{\mathsf{T}}}\Bigg] \qquad \text{(A-35)}\end{aligned}$$

The only missing ingredient is the derivative of $\tilde{\boldsymbol{U}}$ with respect to the mortality schedule for group i.

In the example for *Lomatium bradshawii*, the relationship between $\tilde{\boldsymbol{U}}$ and the mortality vector for a given group is given by the equations in (23), (21), and (60)–(62):

$$\begin{aligned}\tilde{\boldsymbol{U}} &= \mathbb{D}\boldsymbol{K}\mathbb{U}\boldsymbol{K}^{\mathsf{T}} &\text{(A-36)}\\ \mathbb{U} &= \sum_{i=1}^{g}\left(\boldsymbol{L}_i\boldsymbol{U}_i\boldsymbol{Q}_i\right) &\text{(A-37)}\\ \boldsymbol{U}_i &= \boldsymbol{G}_i\boldsymbol{\Sigma}_i &\text{(A-38)}\\ \boldsymbol{\Sigma}_i &= \boldsymbol{I}\circ\left(\mathbf{1}_s\boldsymbol{\sigma}_i^{\mathsf{T}}\right) &\text{(A-39)}\\ \boldsymbol{\sigma}_i &= e^{-\boldsymbol{\mu}_i}. &\text{(A-40)}\end{aligned}$$

Other than Roth's theorem, and the rule for veccing a Hadamard product, we need to know how to differentiate $\boldsymbol{\sigma}_i = e^{-\boldsymbol{\mu}_i}$, which is

$$d\boldsymbol{\sigma}_i = -\mathcal{D}\left(\boldsymbol{\sigma}_i\right)d\boldsymbol{\mu}_i. \qquad \text{(A-41)}$$

Differentiating $\tilde{\boldsymbol{U}}$ with respect to $\mathbb{U}$, differentiating $\mathbb{U}$ with respect to $\boldsymbol{U}_i$, and differentiating $\boldsymbol{U}_i$ with respect to $\boldsymbol{\mu}_i$ gives

$$d\text{vec}\,\tilde{\boldsymbol{U}} = \left(\boldsymbol{K}\otimes\mathbb{D}\boldsymbol{K}\right)\left(\boldsymbol{Q}_i^{\mathsf{T}}\otimes\boldsymbol{L}_i\right)\Big[-\left(\boldsymbol{I}\otimes\boldsymbol{G}_i\right)\mathcal{D}\left(\text{vec}\,\boldsymbol{I}\right)\left(\boldsymbol{I}_s\otimes\mathbf{1}_s\right)\mathcal{D}\left(\boldsymbol{\sigma}_i\right)\Big]d\boldsymbol{\mu}_i. \qquad \text{(A-42)}$$

The derivative of $\tilde{\boldsymbol{U}}$ with respect to $\boldsymbol{\mu}_i$ is obtained from the differential $d\text{vec}\,\tilde{\boldsymbol{U}}$ as

$$\frac{d\text{vec}\,\tilde{\boldsymbol{U}}}{d\boldsymbol{\mu}_i^{\mathsf{T}}} = \left(\boldsymbol{K}\otimes\mathbb{D}\boldsymbol{K}\right)\left(\boldsymbol{Q}_i^{\mathsf{T}}\otimes\boldsymbol{L}_i\right)\Big[-\left(\boldsymbol{I}\otimes\boldsymbol{G}_i\right)\mathcal{D}(\text{vec}\,\boldsymbol{I})\left(\boldsymbol{I}_s\otimes\mathbf{1}_s\right)\mathcal{D}\left(\boldsymbol{\sigma}_i\right)\Big] \qquad \text{(A-43)}$$

(this is the First Identification Theorem of Magnus & Neudecker (1985)). Substituting (A-43) into (A-34) gives the sensitivity of mean lifetime reproductive output with respect to mortality.

Substituting (A-43) into the derivative of the first moment (A-34) and the second moment (A-35) of LRO, and then combining these into

$$\frac{d\tilde{\boldsymbol{v}}}{d\boldsymbol{\mu}_i^{\mathsf{T}}} = \frac{d\tilde{\boldsymbol{\rho}}_2}{d\boldsymbol{\mu}_i^{\mathsf{T}}} - 2\mathcal{D}\left(\tilde{\boldsymbol{\rho}}_1\right)\frac{d\tilde{\boldsymbol{\rho}}_1}{d\boldsymbol{\mu}_i^{\mathsf{T}}}, \tag{A-44}$$

yields the sensitivity of variance in LRO to mortality, as calculated from (42) and (43).

A.3.2 Fertility

When we want to know instead how mean or variance in LRO responds to a change in fertility, all the terms concerning the sensitivity of outcomes with respect to mortality drop out of equations (A-24) and (A-33). These become

$$d\tilde{\boldsymbol{\rho}}_1 = \tilde{\boldsymbol{N}}^{\mathsf{T}}\left[\left(\mathbf{1}^{\mathsf{T}}\otimes\boldsymbol{Z}\right)\boldsymbol{K}_1\mathcal{D}\left(\operatorname{vec}\tilde{\boldsymbol{P}}\right)d\operatorname{vec}\tilde{\boldsymbol{R}}_1\right] \tag{A-45}$$

$$\begin{aligned} d\tilde{\boldsymbol{\rho}}_2 = {} & \tilde{\boldsymbol{N}}^{\mathsf{T}}\Big[\left(\mathbf{1}^{\mathsf{T}}\otimes\boldsymbol{Z}\right)\boldsymbol{K}_1\mathcal{D}\left(\operatorname{vec}\tilde{\boldsymbol{P}}\right)d\operatorname{vec}\tilde{\boldsymbol{R}}_2 \\ & +2\left(\tilde{\boldsymbol{\rho}}_1\otimes\boldsymbol{I}\right)\boldsymbol{K}_2\mathcal{D}\left(\operatorname{vec}\tilde{\boldsymbol{U}}\right)d\operatorname{vec}\hat{\boldsymbol{R}}_1 + \left(\tilde{\boldsymbol{U}}\circ\hat{\boldsymbol{R}}_1\right)^{\mathsf{T}}d\tilde{\boldsymbol{\rho}}_1\Big] \end{aligned} \tag{A-46}$$

In order to reduce the number of matrices we need to differentiate, we can write $\hat{\boldsymbol{R}}_1$ and $\tilde{\boldsymbol{R}}_2$ in terms of $\tilde{\boldsymbol{R}}_1$. Note that rewriting $\tilde{\boldsymbol{R}}_2$ as a function of $\tilde{\boldsymbol{R}}_1$ is not always possible, for example, when the higher moments of fertility are directly obtained from data. In our example we only have information on the first moment, and we make the assumption that fertility is Poisson distributed to obtain

$$\tilde{\boldsymbol{R}}_2 = \tilde{\boldsymbol{R}}_1 + \left(\tilde{\boldsymbol{R}}_1\circ\tilde{\boldsymbol{R}}_1\right). \tag{A-47}$$

Differentiating (A-47) and applying the vec operator gives

$$d\operatorname{vec}\tilde{\boldsymbol{R}}_2 = d\operatorname{vec}\tilde{\boldsymbol{R}}_1 + d\operatorname{vec}\left(\tilde{\boldsymbol{R}}_1\circ\tilde{\boldsymbol{R}}_1\right). \tag{A-48}$$

Applying the rule for differentiating Hadamard products, this becomes

$$d\operatorname{vec}\tilde{\boldsymbol{R}}_2 = d\operatorname{vec}\tilde{\boldsymbol{R}}_1 + 2\mathcal{D}\left(\operatorname{vec}\tilde{\boldsymbol{R}}_1\right)d\operatorname{vec}\tilde{\boldsymbol{R}}_1, \tag{A-49}$$

which can be written as

$$d\operatorname{vec}\tilde{\boldsymbol{R}}_2 = \left(\boldsymbol{I}_{(gs+1)^2} + 2\mathcal{D}\left(\operatorname{vec}\tilde{\boldsymbol{R}}_1\right)\right)d\operatorname{vec}\tilde{\boldsymbol{R}}_1. \tag{A-50}$$

$\hat{\boldsymbol{R}}_1$ is $\tilde{\boldsymbol{R}}_1$ with the absorbing states removed;

$$\hat{\boldsymbol{R}}_1 = \boldsymbol{Z}\tilde{\boldsymbol{R}}_1\boldsymbol{Z}^{\mathsf{T}}. \tag{A-51}$$

Differentiating (A-51), and applying the vec operator yields

$$d\operatorname{vec}\hat{\boldsymbol{R}}_1 = \operatorname{vec}\left(\boldsymbol{Z}d\tilde{\boldsymbol{R}}_1\boldsymbol{Z}^{\mathsf{T}}\right), \tag{A-52}$$

which, following Roth's theorem, gives

$$d\operatorname{vec}\hat{\boldsymbol{R}}_1 = \left(\boldsymbol{Z}\otimes\boldsymbol{Z}\right)d\operatorname{vec}\tilde{\boldsymbol{R}}_1. \tag{A-53}$$

Now we can write equations (A-45) and (A-46) in terms of the derivatives of $\tilde{\boldsymbol{\rho}}_1$, $\tilde{\boldsymbol{\rho}}_2$, and $\tilde{\boldsymbol{R}}_1$;

$$\frac{d\tilde{\boldsymbol{\rho}}_1}{d\boldsymbol{f}_i^{\mathsf{T}}} = \tilde{\boldsymbol{N}}^{\mathsf{T}}\left(\mathbf{1}^{\mathsf{T}}\otimes\boldsymbol{Z}\right)\boldsymbol{K}_1\mathcal{D}\left(\operatorname{vec}\tilde{\boldsymbol{P}}\right)\frac{d\operatorname{vec}\tilde{\boldsymbol{R}}_1}{d\boldsymbol{f}_i^{\mathsf{T}}} \tag{A-54}$$

$$\begin{aligned} \frac{d\tilde{\boldsymbol{\rho}}_2}{d\boldsymbol{f}_i^{\mathsf{T}}} = {} & \tilde{\boldsymbol{N}}^{\mathsf{T}}\Bigg[\left(\mathbf{1}^{\mathsf{T}}\otimes\boldsymbol{Z}\right)\boldsymbol{K}_1\mathcal{D}\left(\operatorname{vec}\tilde{\boldsymbol{P}}\right)\left(\boldsymbol{I}_{(gs+1)^2} + 2\mathcal{D}\left(\operatorname{vec}\tilde{\boldsymbol{R}}_1\right)\right)\frac{d\operatorname{vec}\tilde{\boldsymbol{R}}_1}{d\boldsymbol{f}_i^{\mathsf{T}}} \\ & +2\left(\tilde{\boldsymbol{\rho}}_1\otimes\boldsymbol{I}\right)\boldsymbol{K}_2\mathcal{D}\left(\operatorname{vec}\tilde{\boldsymbol{U}}\right)\left(\boldsymbol{Z}\otimes\boldsymbol{Z}\right)\frac{d\operatorname{vec}\tilde{\boldsymbol{R}}_1}{d\boldsymbol{f}_i^{\mathsf{T}}} + \left(\tilde{\boldsymbol{U}}\circ\hat{\boldsymbol{R}}_1\right)^{\mathsf{T}}\frac{d\tilde{\boldsymbol{\rho}}_1}{d\boldsymbol{f}_i^{\mathsf{T}}}\Bigg]. \end{aligned} \tag{A-55}$$

The matrix $\tilde{\boldsymbol{R}}_1$ is a function of $\boldsymbol{f}_1$ through the following set of equations;

$$\tilde{\boldsymbol{R}}_1 = \mathbf{1}_{gs+1}\tilde{\boldsymbol{f}}^{\mathsf{T}}\boldsymbol{Z} \tag{A-56}$$

$$\tilde{\boldsymbol{f}} = \boldsymbol{K}\sum_{i=1}^{g}\left(\boldsymbol{L}_i\boldsymbol{f}_i\right). \tag{A-57}$$

The derivative of $\tilde{\boldsymbol{R}}_1$ with respect to $\boldsymbol{f}_i$ is obtained through the usual steps; differentiation, applying the vec operator and then Roth's theorem if necessary;

$$d\text{vec}\,\tilde{\boldsymbol{R}}_1 = \left(\boldsymbol{Z}^{\mathsf{T}} \otimes \mathbf{1}_{gs+1}\right)\boldsymbol{K}\boldsymbol{L}_i d\boldsymbol{f}_i. \tag{A-58}$$

Invoking the First Identification Theorem gives

$$\frac{d\text{vec}\,\tilde{\boldsymbol{R}}_1}{d\boldsymbol{f}_i^{\mathsf{T}}} = \left(\boldsymbol{Z}^{\mathsf{T}} \otimes \mathbf{1}_{gs+1}\right)\boldsymbol{K}\boldsymbol{L}_i. \tag{A-59}$$

Combining (A-59), (A-54), and (A-55) provides the sensitivity of mean and variance in LRO to fertility.

A.3.3 By your powers combined...

Presenting sensitivity analyses of matrix expressions with long chains of dependency (Figure 1) confronts the researcher with a tricky decision. Whether we present it as a long chain rule for which we collect the individual parts (as we do in the main text), or just start somewhere and 'differentiate down' (as we did here), it is a challenge to keep track of all the parts of the equations. Did this matrix lose a transpose? Is this supposed to be $\boldsymbol{K}_1$ or $\boldsymbol{K}_2$? Are all the dimensions correct? Once you have the correct expression (and have successfully implemented it in MATLAB or your matrix language of choice), it is tempting to show that final equation you worked so hard to obtain. However, the final product, for example, sensitivity of variance in LRO to mortality for *Lomatium*, might look like this:

$$\begin{aligned}
\frac{d\tilde{\boldsymbol{v}}}{d\boldsymbol{\mu}_i^{\mathsf{T}}} = \; & \tilde{\boldsymbol{N}}^{\mathsf{T}}\Big\{(\mathbf{1}^{\mathsf{T}}\otimes\boldsymbol{Z})\boldsymbol{K}_1\mathcal{D}(\text{vec}\,\tilde{\boldsymbol{R}}_2)\left[\boldsymbol{C}_1 - \boldsymbol{C}_2\left(\boldsymbol{I}_{gs}\otimes\mathbf{1}_{gs}^{\mathsf{T}}\right)\right] \\
& +2(\tilde{\boldsymbol{\rho}}_1\otimes\boldsymbol{I})\boldsymbol{K}_2\mathcal{D}(\text{vec}\,\hat{\boldsymbol{R}}_1)\Big\} \\
& \times(\boldsymbol{K}\otimes\mathbb{D}\boldsymbol{K})(\boldsymbol{Q}_i^{\mathsf{T}}\otimes\boldsymbol{L}_i)\left[-(\boldsymbol{I}\otimes\boldsymbol{G}_i)\,\mathcal{D}(\text{vec}\,\boldsymbol{I})\,(\boldsymbol{I}_s\otimes\mathbf{1}_s)\,\mathcal{D}(\boldsymbol{\sigma}_i)\right] \\
& +\tilde{\boldsymbol{N}}^{\mathsf{T}}\left(\tilde{\boldsymbol{U}}\circ\hat{\boldsymbol{R}}_1\right)^{\mathsf{T}}\tilde{\boldsymbol{N}}^{\mathsf{T}}\Big\{(\mathbf{1}^{\mathsf{T}}\otimes\boldsymbol{Z})\boldsymbol{K}_1\mathcal{D}(\text{vec}\,\tilde{\boldsymbol{R}}_1)\left[\boldsymbol{C}_1 - \boldsymbol{C}_2\left(\boldsymbol{I}_{gs}\otimes\mathbf{1}_{gs}^{\mathsf{T}}\right)\right] \\
& +\left(\tilde{\boldsymbol{\rho}}_1^{\mathsf{T}}\otimes\boldsymbol{I}\right)\boldsymbol{K}_2\Big\}(\boldsymbol{K}\otimes\mathbb{D}\boldsymbol{K})(\boldsymbol{Q}_i^{\mathsf{T}}\otimes\boldsymbol{L}_i)\left[-(\boldsymbol{I}\otimes\boldsymbol{G}_i)\,\mathcal{D}(\text{vec}\,\boldsymbol{I})\,(\boldsymbol{I}_s\otimes\mathbf{1}_s)\,\mathcal{D}(\boldsymbol{\sigma}_i)\right] \\
& +\tilde{\boldsymbol{N}}^{\mathsf{T}}(\tilde{\boldsymbol{\rho}}_2^{\mathsf{T}}\otimes\boldsymbol{I})\boldsymbol{K}_2(\boldsymbol{K}\otimes\mathbb{D}\boldsymbol{K})(\boldsymbol{Q}_i^{\mathsf{T}}\otimes\boldsymbol{L}_i)\left[-(\boldsymbol{I}\otimes\boldsymbol{G}_i)\,\mathcal{D}(\text{vec}\,\boldsymbol{I})\,(\boldsymbol{I}_s\otimes\mathbf{1}_s)\,\mathcal{D}(\boldsymbol{\sigma}_i)\right] \\
& -2\mathcal{D}(\tilde{\boldsymbol{\rho}}_1)\tilde{\boldsymbol{N}}^{\mathsf{T}}\Big\{(\mathbf{1}^{\mathsf{T}}\otimes\boldsymbol{Z})\boldsymbol{K}_1\mathcal{D}(\text{vec}\,\tilde{\boldsymbol{R}}_1)\left[\boldsymbol{C}_1 - \boldsymbol{C}_2\left(\boldsymbol{I}_{gs}\otimes\mathbf{1}_{gs}^{\mathsf{T}}\right)\right] + (\tilde{\boldsymbol{\rho}}_1^{\mathsf{T}}\otimes\boldsymbol{I})\boldsymbol{K}_2\Big\} \\
& \times(\boldsymbol{K}\otimes\mathbb{D}\boldsymbol{K})(\boldsymbol{Q}_i^{\mathsf{T}}\otimes\boldsymbol{L}_i)\left[-(\boldsymbol{I}\otimes\boldsymbol{G}_i)\,\mathcal{D}(\text{vec}\,\boldsymbol{I})\,(\boldsymbol{I}_s\otimes\mathbf{1}_s)\,\mathcal{D}(\boldsymbol{\sigma}_i)\right]
\end{aligned} \tag{A-60}$$

This expression might be the final product, and it might be impressive, but it does not provide any insight (nor does it spark joy, even in us). It is a solution for the sensitivity of variance in LRO to mortality, but an attempt to code it as a single expression is likely to produce errors. Programming each piece of the chain of derivatives is a better solution. In addition, the parts of the calculation can be useful to other investigators performing sensitivity analysis even if

their model differs in some components. By presenting the chain of consecutive sensitivities, it is easier for other investigators to identify where their analyses might differ.

This expression,

$$\frac{d\tilde{\boldsymbol{v}}}{d\boldsymbol{\mu}_i^{\mathsf{T}}} = \left(\frac{d\tilde{\boldsymbol{v}}}{d\mathrm{vec}^{\mathsf{T}}\tilde{\boldsymbol{U}}}\right)\left(\frac{d\mathrm{vec}\,\tilde{\boldsymbol{U}}}{d\mathrm{vec}^{\mathsf{T}}\mathbb{U}}\right)\left(\frac{d\mathrm{vec}\,\mathbb{U}}{d\mathrm{vec}^{\mathsf{T}}\boldsymbol{U}_i}\right)\left(\frac{d\mathrm{vec}\,\boldsymbol{U}_i}{d\boldsymbol{\mu}_i^{\mathsf{T}}}\right), \tag{A-61}$$

together with expressions for each of the derivatives, contains the same information as (A-60) with the advantage that it shows the steps required to get the solution. Some matrix calculus is required to get there, and although differentiating matrices with respect to vectors can seem daunting at first, it requires only a few main steps (just 6 according to Caswell (2019)). Matrix calculus is your friend, not just as a technique for sensitivity analysis, but also as a way to present it. If the main text of our paper did not convince you of this, maybe this little appendix has.

References

Caswell, H. (2019). *Sensitivity Analysis: Matrix Methods in Demography and Ecology*. Demographic Research Monographs. Springer International Publishing.

Caswell, H. & van Daalen, S. F. (2016). A note on the vec operator applied to unbalanced block-structured matrices. *Journal of Applied Mathematics*, *2016*(Article ID 4590817), 1–3.

Magnus, J. R. & Neudecker, H. (1985). Matrix differential calculus with applications to simple, Hadamard, and Kronecker products. *Journal of Mathematical Psychology*, *29*(4), 474–492.

van Daalen, S. F. & Caswell, H. (2017). Lifetime reproductive output: individual stochasticity, variance, and sensitivity analysis. *Theoretical Ecology*, *10*(3), 355–374.
